# NKG2D blockade impairs tissue-resident memory T cell accumulation and reduces chronic lung allograft dysfunction

**DOI:** 10.1172/jci.insight.184048

**Published:** 2025-02-24

**Authors:** Kaveh Moghbeli, Madeline A. Lipp, Marta Bueno, Andrew Craig, Michelle Rojas, Minahal Abbas, Zachary I. Lakkis, Byron Chuan, John Sembrat, Kentaro Noda, Daniel J. Kass, Kong Chen, Li Fan, Tim Oury, Zihe Zhou, Xingan Wang, John F. McDyer, Oliver Eickelberg, Mark E. Snyder

**Affiliations:** 1 Department of Medicine,; 2 Department of Pharmacy,; 3 Department of Cardiothoracic Surgery, and; 4 Department of Pathology, University of Pittsburgh, Pittsburgh, Pennsylvania, USA.; 5 Starzl Transplantation Institute, Pittsburgh, Pennsylvania, USA.; 6 Department of Immunology, University of Pittsburgh, Pittsburgh, Pennsylvania, USA.

**Keywords:** Immunology, Transplantation, Cellular immune response, Organ transplantation, T cells

## Abstract

Chronic lung allograft dysfunction (CLAD) substantially limits long-term survival following lung transplantation. To identify potential targets for CLAD prevention, T cells from explanted CLAD lungs and lung-draining lymph nodes, as well as diseased and nondiseased controls were isolated and single-cell RNA sequencing and TCR sequencing were performed. TCR sequencing revealed a clonally expanded population of CD8^+^ tissue-resident memory T cells (TRMs) with high cytotoxic potential, including upregulation of *KLRK1*, encoding the co-receptor NKG2D. These cytotoxic CD8^+^ TRMs accumulated around the CLAD airways and had a 100-fold increase in clonal overlap with lung-draining lymph nodes when compared with non-CLAD lungs. Using a murine model of orthotopic lung transplantation, we confirmed that cytotoxic CD8^+^ TRM accumulation was due to chronic rejection and not transplantation alone. Furthermore, blocking NKG2D in vivo attenuated the airway remodeling following transplantation and diminished airway accumulation of CD8^+^ T cells. Our findings support NKG2D as a potential therapeutic target for CLAD, affecting cytotoxic CD8^+^ TRM accumulation.

## Abstract 2

In-vivo NKG2D blockade attenuates airway damage caused by cytotoxic CD8^+^ tissue resident memory T cells accumulating around the airways during chronic lung allograft dysfunction.

## Graphical abstract

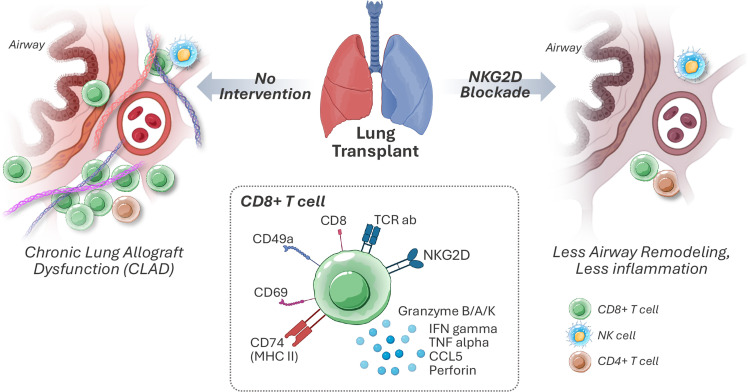

## Introduction

Lung transplantation remains the only therapeutic option for many patients with end-stage lung diseases such as chronic obstructive pulmonary disease and pulmonary fibrosis. However, lung transplant recipient (LTR) survival remains poor, with a median survival of only 6 years, compared with 12 or more years for heart or kidney transplantation (1–3). Long-term survival is most heavily impacted by the development of chronic lung allograft dysfunction (CLAD), which presents as either a progressive narrowing of the small airways leading to progressive obstructive ventilatory defect (bronchiolitis obliterans syndrome) or a progressive peripheral fibrotic process leading to a restrictive ventilatory defect (restrictive CLAD). CLAD is associated with marked morbidity and mortality in LTRs, accounting for more than 40% of deaths after the first posttransplant year. While T cell–mediated alloimmunity is believed to be the primary driver of CLAD, mechanisms remain incompletely characterized and effective therapy to prevent or reverse CLAD remains a major unmet need in lung transplantation (4, 5).

Following lung transplantation, there is both persistence of donor-derived mucosal T cells and recruitment to the allograft of recipient-derived T cells that take up long-term residency, primarily in and around the airways as tissue-resident memory T cells (TRMs) (6). The degree of allograft chimerism (coexistence of T cells from both donor and recipient) can be impacted by both primary allograft dysfunction and acute cellular rejection (ACR), which appear to lead to a more rapid turnover of allograft TRMs (7), suggesting the recruitment and retention of allograft-specific T cells to the lungs. This is supported by the finding that clonally expanded CD8^+^ T cells found within the perivascular space of the allograft at the time of ACR persist after treatment with high-dose systemic steroids and migrate to the airways, providing a putative link between ACR and future airway damage. This damage may occur via the upregulation of donor major histocompatibility complex (MHC) by basal cells, promoting airway damage by alloreactive, cytotoxic CD8^+^ T cells (5). However, it remains unknown whether these cytotoxic CD8^+^ T cells are sustained as airway-associated, recipient-derived, alloreactive TRMs and whether they can be targeted to attenuate injury.

We sought to characterize the origin (donor vs. recipient), phenotype, T cell receptor (TCR) diversity, and function of T cells found residing within the lung allograft and hilar lymph nodes (HLNs) at the time of CLAD and compare these with lungs and HLNs from organ donors without underlying lung disease and explants from idiopathic pulmonary fibrosis (IPF). In so doing, we identified a population of clonally expanded, NKG2D^+^ CD8^+^ T cells with a transcriptomic and protein signature suggestive of cytotoxic activity. We found that CLAD HLNs were of donor origin, but repopulated with recipient immune cells, including a significantly increased clonal overlap with lung TRMs. Using a murine orthotopic lung transplant (OLT) model of CLAD, we confirmed that the accumulation of alloreactive TRMs was due to CLAD, not transplantation, and that the systemic delivery of an NKG2D-blocking antibody could partially reverse the pathophysiology associated with CLAD. Taken together, these findings highlight that CLAD is associated with the accumulation of airway-centered, cytotoxic, NKG2D^+^CD8^+^ TRMs and that systemic NKG2D is a potential target to prevent their accumulation after transplantation.

## Results

### Lungs and lung-draining lymph nodes in patients with CLAD are repopulated with a predominantly CD8^+^ recipient-derived TRM population.

**Figure 1:**
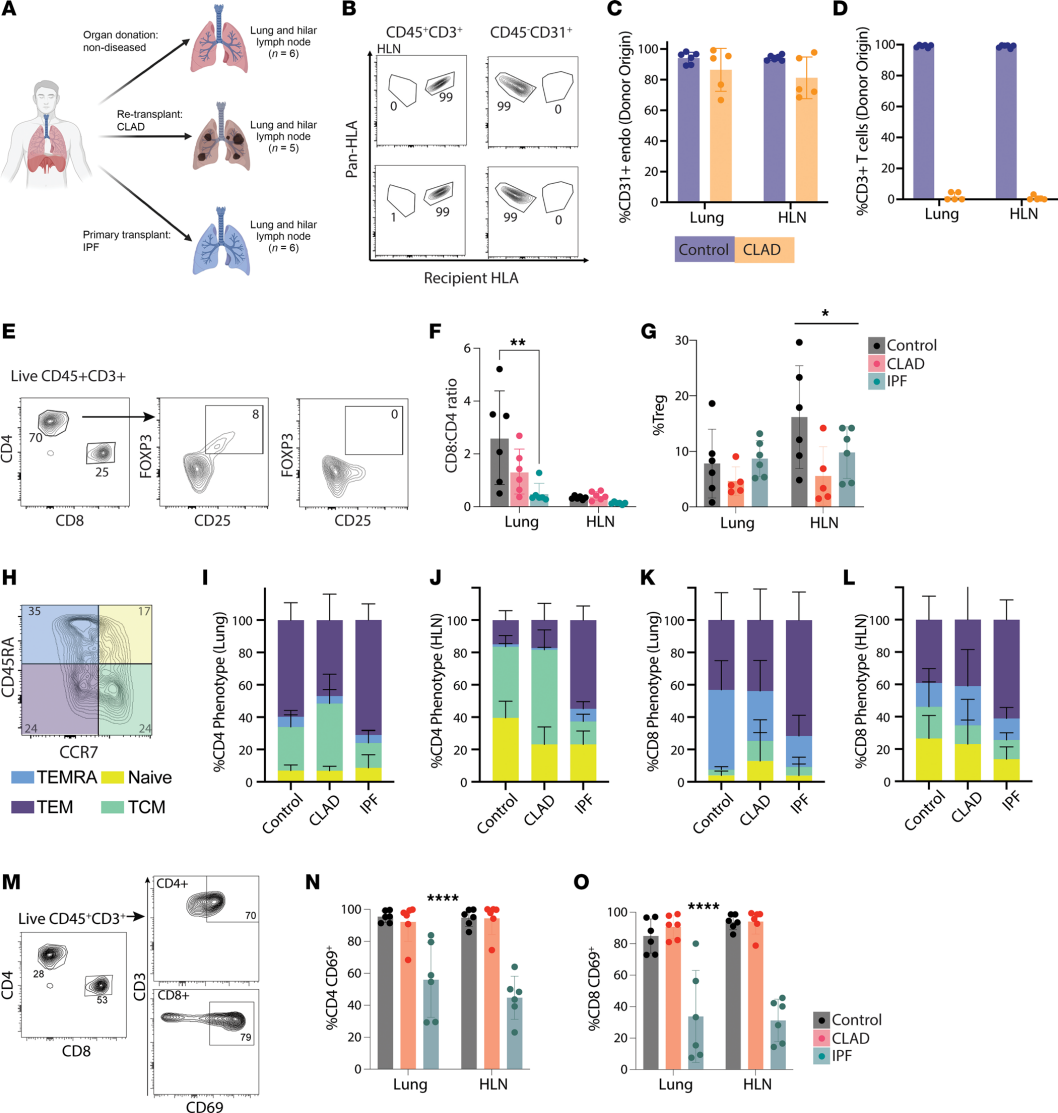
Lung and HLN memory T cell subsets from patients with CLAD, IPF, and without chronic lung disease. (**A**) Schematic representation of cell sources for all human ex vivo data. (**B**) Representative flow plots of HLN cells stained for CD45, CD3, CD8, CD31, T1a, and a combination of either donor- or recipient-specific and pan-positive HLA to determine cell origin. (**C**) Mean proportion of donor HLA–positive endothelial cells from CLAD (orange) and negative control from nondiseased HLNs (blue) (*n* = 5). (**D**) Mean proportion of donor HLA–positive T cells from CLAD HLNs (orange) and negative control from control HLNs (blue) (*n* = 6). (**E**) Representative flow plots for CD4^+^, CD8^+^, and CD4^+^FOXP3^+^CD25^+^ T cells. (**F**) Cumulative data showing the ratio of CD8^+^ to CD4^+^ CD3^+^ T cells found in the lung and HLNs across conditions (*n* = 6 Control, *n* = 6 CLAD, *n* = 6 IPF). (**G**) Proportion of FOXP3^+^CD25^+^ (Treg) CD4^+^ T cells within lung and HLNs (*n* = 6 Control, *n* = 5 CLAD, *n* = 6 IPF). (**H**) Representative flow plot of memory T cell subsets identified through expression of CD45RA and CCR7, highlighting effector memory T cells (TEM: CD45RA^–^CCR7^–^), terminally differentiated effector T cells (TEMRA: CD45RA^+^CCR7^–^), Naive (CD45RA^+^CCR7^+^), and central memory T cells (TCM: CD45RA^–^CCR7^+^). (**I**–**L**) Mean proportion of memory subsets of (**I**) lung CD4^+^ T cells, (**J**) HLN CD4^+^ T cells, (**K**) lung CD8^+^ T cells, and (**L**) HLN CD8^+^ T cells. (**M**) Representative flow plot of CD4^+^ and CD8^+^ tissue-resident memory (TRM) (CD69^+^) T cell subsets. (**N**) Mean proportion of CD4^+^ TRMs among all CD4^+^ T cells. (**O**) Mean proportion of CD8^+^ TRMs among all CD8^+^ T cells. *P < 0.05, **P < 0.01, ***P < 0.001 (FDR-adjusted) by 2-way ANOVA, displaying column comparisons between conditions). All bar charts show mean ± SD.

Explanted lungs and HLNs from patients with CLAD undergoing re-transplantation underwent mechanical and enzymatic digestion to obtain a single-cell suspension for flow cytometric analysis. Lungs from deceased donors with IPF were utilized as a diseased comparator (Figure 1A and Supplemental Figure 1; supplemental material available online with this article; https://doi.org/10.1172/jci.insight.184048DS1). HLN stromal cells, including endothelial (CD45^–^CD31^+^) and lymphatic (CD45^–^T1a^+^), as well as endothelial cells from explanted lung allografts were universally positive for donor-specific human leukocyte antigen (HLA) and negative for recipient-specific HLA, indicating that HLNs were of donor origin and not a result of posttransplant de novo lymphoid organogenesis (Figure 1, B and C, and Supplemental Figure 2). In contrast, nearly all lymphocytes (T and B cells) from HLNs stained positive for recipient HLA, indicating recipient origin (Figure 1D). Most T cells found in the HLNs were CD4^+^, with no significant differences in proportion across all conditions (Figure 1F). There was an increased proportion of CD8^+^ T cells found in both the lung of control and CLAD lungs compared with those from IPF (*P* < 0.05 for control vs. IPF) (Figure 1G). In addition, a trend toward reduced Treg cell proportions (CD4^+^CD127^lo^CD25^hi^FOXP3^+^) was observed in CLAD lungs and HLNs compared with both IPF and control groups (Figure 1, E and G).

Among the CD4^+^ T cells in the lungs and HLNs, an increased proportion of T central memory (TCM) cells (CCR7^+^CD45RA^–^) was observed in CLAD, compared with control and IPF; however, this did not reach statistical significance (Figure 1, H–J). Among CD8^+^ T cells, an increased proportion of T effector memory cells re-expressing CD45RA (TEMRA) (CCR7^–^CD45RA^+^) was seen in the HLNs from patients with CLAD compared with control HLNs; however, this trend was reversed in the lungs, with neither reaching statistical significance (Figure 1, K and L). Among the memory compartments, the only statistically significant difference was an increased proportion of both CD4^+^ TEMRA and effector memory T cells (TEM) cells in IPF HLNs compared with the respective compartments in control HLNs (*P* < 0.05).

TRMs were identified based on expression of the canonical TRM cell surface marker CD69 (Figure 1M) (8). No statistically significant differences in CD4^+^ TRM or CD8^+^ TRM proportions were observed between CLAD and control in either the lungs or HLNs. However, decreased proportions of both CD4^+^ and CD8^+^ TRMs were observed in IPF lungs and HLNs compared with both CLAD and control (Figure 1, N and O).

**Figure 2:**
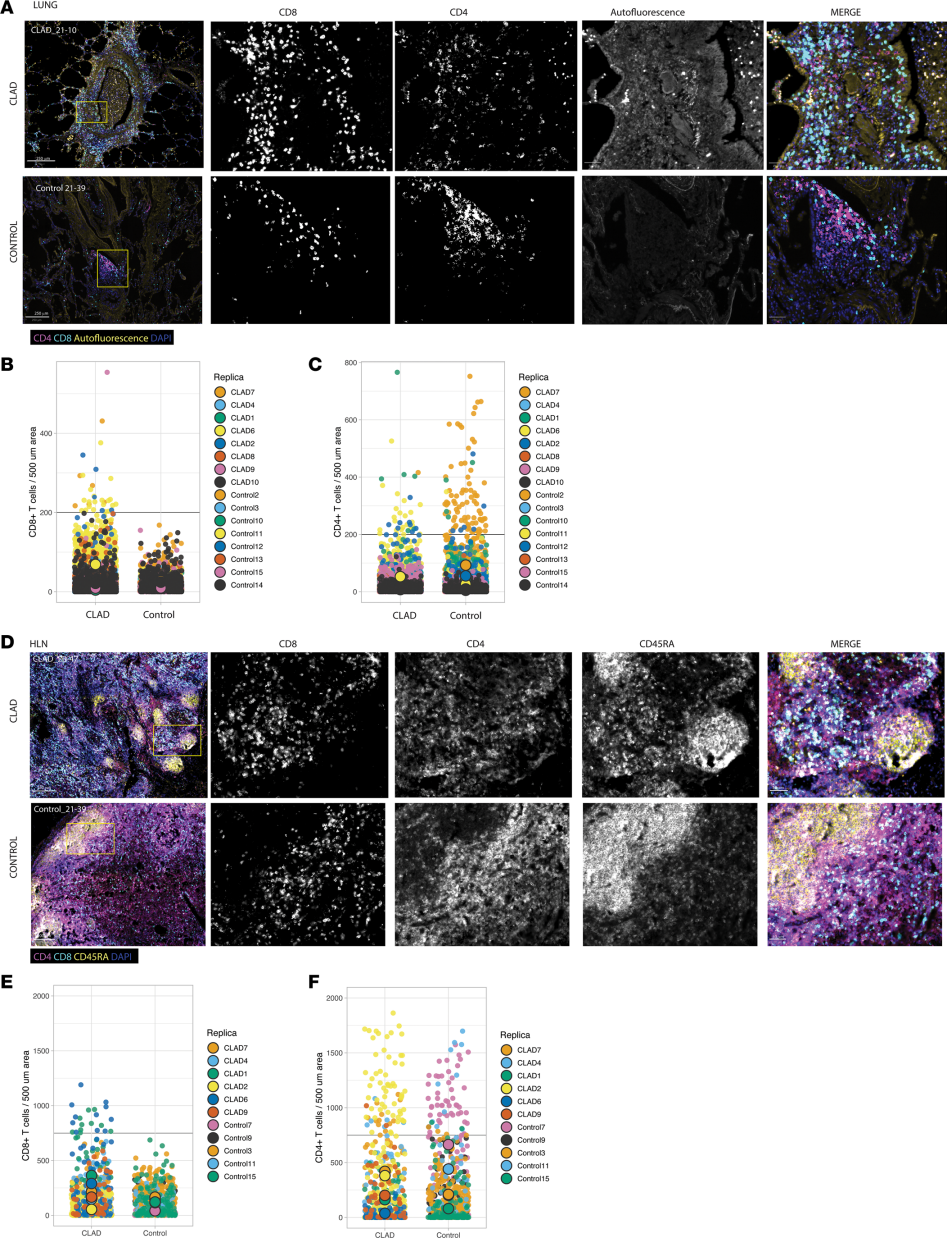
CLAD lungs and HLNs contain increased clusters of CD8^+^ T cells. (**A**) Representative immunofluorescence images of lungs from CLAD (top row) and control (bottom row). Scale bars: 250 μm (6 left images) and 50 μm (4 right images). (**B** and **C**) Normalized CD8^+^ (**B**) and CD4^+^ (**C**) T cell counts per 500 mm^2^ in lung CLAD and control samples; dotted line represents threshold of 180 cells per mm^2^ of lung. (**D**) Normalized CD8^+^ (top) and CD4^+^ (bottom) T cell counts per 500 mm^2^ in HLN CLAD and control samples. (**D**) Representative HLN immunofluorescence images from CLAD (top row) and 1 control (bottom row). Scale bars: 250 μm (8 left images) and 50 μm (2 right images). (**E** and **F**) Normalized CD8^+^ (**E**) and CD4^+^ (**F**) T cell counts per 500 mm^2^ in HLN CLAD and control samples; dotted line represents threshold of 500 cells per mm^2^.

We next performed immunofluorescence imaging of explanted CLAD and control lungs to quantify T cell content and explore differential patterns of T cell accumulation. Subjectively, we found that most CD8^+^ and CD4^+^ T cells were found in or around the small airways, with CD8^+^ T cells more frequently found in the intraepithelial and subepithelial compartment and CD4^+^ T cells more often found in the peribronchial and peribronchiolar regions; importantly, we found that in CLAD lungs, these clusters of airway-centered T cells were greater in number in CLAD lungs when compared with control (Figure 2A). When systematically quantifying T cell composition by unit area, we found a clear pattern of large clusters of CD8^+^ T cells found only in the CLAD lungs (Figure 2B), whereas large clusters of CD4^+^ T cells were identified in both the CLAD and control lungs at similar frequencies (Figure 2C). We found a similar trend in T cell proportions in the HLNs of CLAD and control lungs. Subjectively, we identified smaller, more numerous follicles in the HLNs of CLAD lungs when compared with control (Figure 2D). HLNs from CLAD had larger clusters of CD8^+^ T cells when compared with control (Figure 2E) and a similar content of CD4^+^ T cell clusters (Figure 2F). In keeping with our findings that CD8^+^ T cells were mainly localized to the airways, we found that most CD8^+^ T cells colocalized with Club cell 10-kDa protein (CC10), a marker for club cells, but we found no consistent relationship between the degree of CD8^+^ T cell accumulation and the degree of CC10 staining (Supplemental Figure 3A). There were some airways with large clusters of CD8^+^ T cells where CC10 staining appeared diminished with abnormal epithelial cell morphology, and those where CC10 staining remained normal with intact columnar epithelial cells. Subjectively, IPF lungs showed a pattern of airway CD8^+^ T cell localization similar to that of control lungs, but unlike CLAD or control, IPF lungs displayed more clusters of parenchyma-based CD8^+^ T cells, far removed from CC10-positive cells (Supplemental Figure 3B). These findings support the assertion that in CLAD, CD8^+^ T cells accumulate in large numbers in and around the airways and that the replacement of donor cells in the HLNs results in the preferential accumulation of recipient CD8^+^ TRMs.

### CLAD T cell repertoire demonstrates oligoclonal expansion overlapping between the lung and HLN.

**Figure 3:**
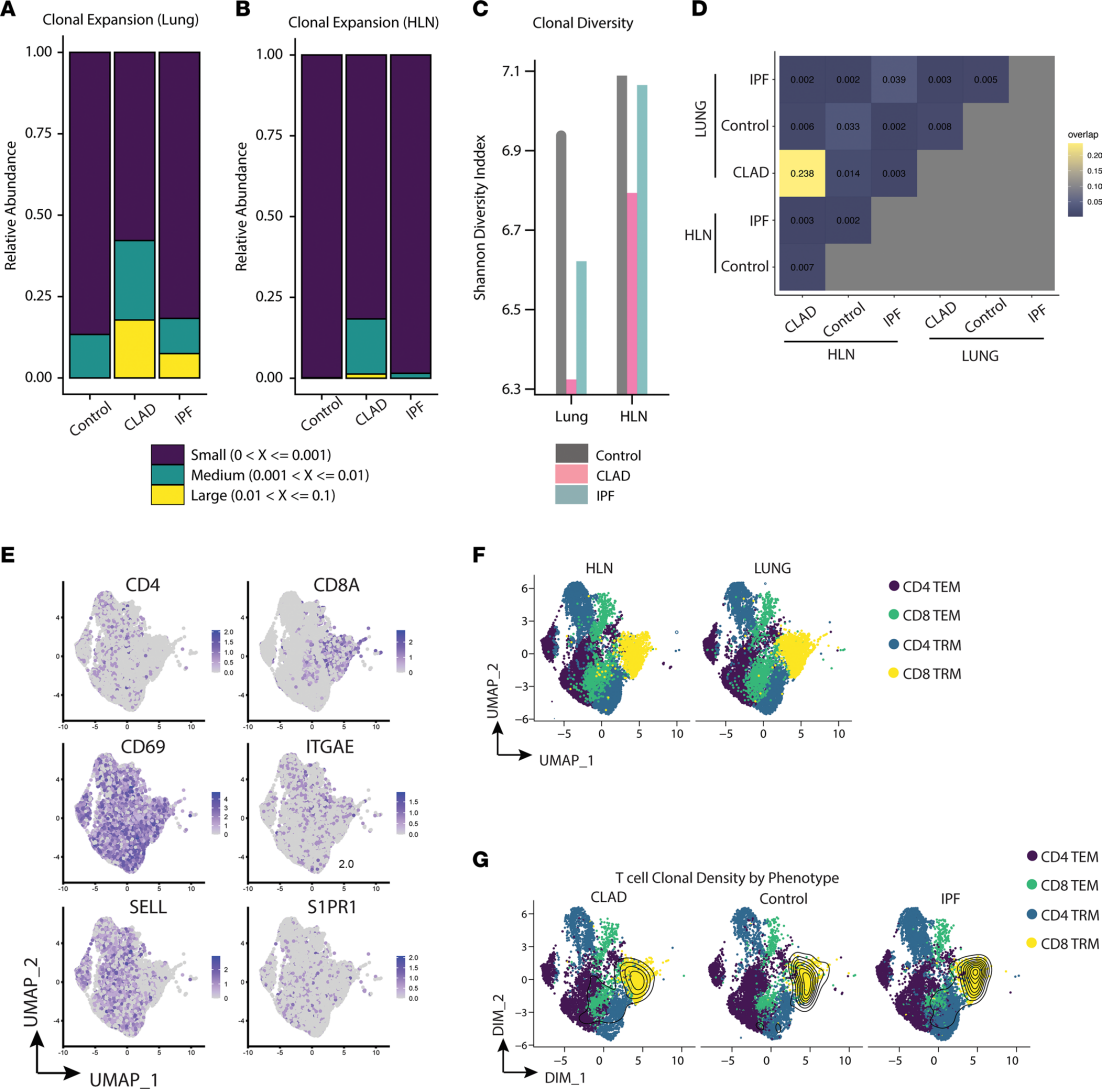
T cells from CLAD lungs and HLNs demonstrate increased clonal expansion of CD8^+^ TRMs, with a 100-fold increase in clonal overlap in CLAD. (**A** and **B**) Proportions of clonally expanded T cell populations from lungs and HLNs. (**C**) Lung and HLN T cell clonal diversity. (**D**) Proportion of overlapping T cell clonality between lung and HLN T cell populations. (**E**) Uniform manifold approximation and projection (UMAP) of T cell marker genes. (**F**) UMAP demonstrating cellular abundance of each T cell subset by anatomic location. (**G**) Density of clonal expansion by T cell subset and disease. EM, effector memory; TRM, tissue-resident memory.

Following mechanical and enzymatic digestion of lung and HLN samples, T cells were sorted for paired single-cell RNA and TCR sequencing. We found an increased proportion of clonally expanded T cells from CLAD lungs compared with both IPF and control (Figure 3A). This finding persisted to a smaller degree in HLNs from patients with CLAD (Figure 3B). In addition to substantial clonal expansion, the T cell repertoire from both CLAD lungs and HLNs demonstrated markedly decreased clonal diversity compared with both control and IPF (Figure 3C). Significant T cell clonal overlap was only seen between lungs and HLNs from CLAD, with over 20% clonal overlap, as opposed to IPF and control lungs which had, at most, 2.6% clonal overlap between lungs and HLNs (Figure 3D). We next explored gene expression between disease states. Following quality control measures and normalization, dimensionality reduction was performed using uniform manifold approximation and projection (UMAP). Rather than unsupervised clustering to identify distinct transcriptional states of T cells, we chose to explore population differences based on biological knowledge of transcripts known to account for major phenotypes of CD4^+^ and CD8^+^ T cells. Four predominant phenotypic groupings — CD4^+^ effector memory (EM), CD8^+^ EM, CD4^+^ TRM, and CD8^+^ TRM — were identified among the combined T cells from all disease states and anatomic locations based on expression of *CD4*, *CD8A*, *CD69*, *ITGAE*, *SELL*, and *S1PR1* (Figure 3, E and F). Combining our RNA and TCR datasets, we then projected our findings of clonal expansion upon this UMAP, with results showing that clonal expansion was primarily focused within the CD8^+^ TRM subset found in both the lungs and HLNs (Figure 3G). We found similar results with traditional unsupervised clustering, with the vast majority of clonal expansion isolated to 1 cluster of CD8^+^ T cells (Supplemental Figure 4).

### CLAD T cells are associated with a cytotoxic cytokine and serine protease profile.

**Figure 4:**
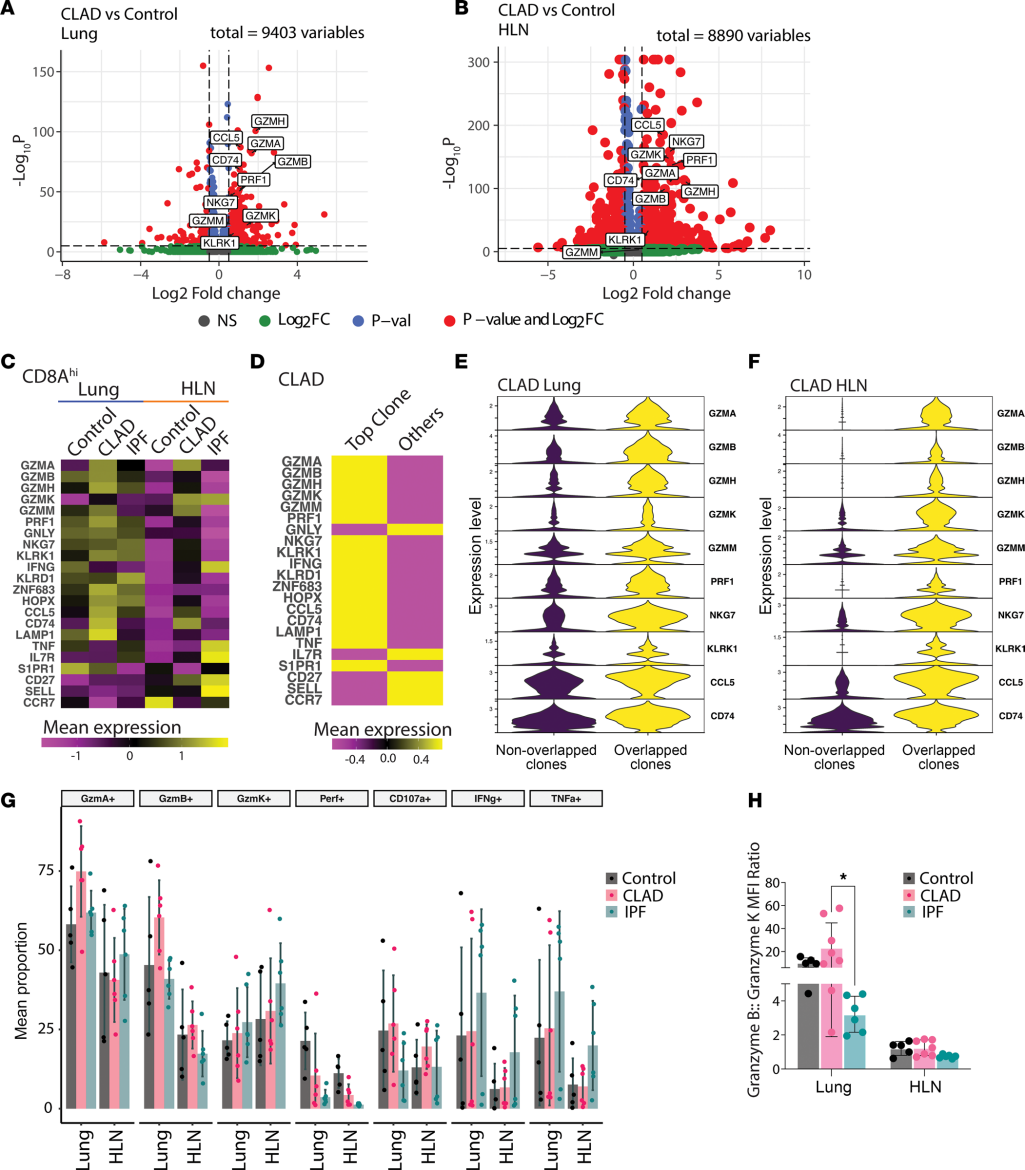
CLAD T cells are associated with a cytotoxic cytokine profile. (**A** and **B**) Differentially expressed genes between patients with CLAD and nondiseased control patient for lung (**A**) and HLN (**B**) T cells. (**C**) Mean normalized CD8^+^ T cell expression of genes associated with cytotoxic effector function by disease and anatomic location. (**D**) Mean normalized T cell expression of genes associated with cytotoxic effector function, comparing T cells comprising the most clonally expanded T cells from patients with CLAD (comprising top 10% of clonally expanded cells for that patient) and all other remaining CLAD T cells. (**E** and **F**) Expression of genes associated with cytotoxic effector function within T cells in overlapping and nonoverlapping clonal populations between the lungs (**E**) and HLNs (**F**) in CLAD. (**G**) Mean proportion of CD8^+^ T cells expressing cytotoxic effector proteins, cytokines, and markers of activation by disease and anatomic location. (**H**) Ratio of granzyme B to granzyme K mean MFI for lung and HLN CD8^+^ T cells (*n* = 5 control, *n* = 8 CLAD, *n* = 6 IPF). *P < 0.05 by Wilcoxon’s rank-sum test.

Differential gene expression analysis from single-cell RNA sequencing comparing CLAD with control lungs and HLNs identified upregulation of genes associated cytotoxicity (*GZMA*, *GZMB*, *GZMK*, *GZMH*, *PRF1*, *GNLY*, *NKG7*, and *KLRK1*) antigen presentation (*CD73*) and chemotaxis (*CCL5*), among others (Figure 4, A and B). When exploring mean expression of genes differentially expressed between CLAD, IPF, and control lungs and HLNs, we found a consistent pattern of CLAD lungs and HLNs showing relative upregulation of genes encoding cytolytic enzymes (*GZMA*, *GZMB*, *GZMH*, and *GZMM*) as well as pore formation for delivery of these enzymes (*PRF1*) and cytolytic granule degranulation (*LAMP1*). Interestingly, *GZMK* expression was highest in the HLNs from CLAD and IPF explants. CD8^+^ T cells from CLAD lungs and HLNs also showed upregulation of cell surface markers associated with both NK cells and cytotoxic CD8^+^ T cells (*KLRD1*, *NKG7*, and *KLRK1*) (Figure 4C). A similar pattern of upregulation of genes associated with cytotoxicity was seen among the CD4^+^ T cells found in the lungs and HLNs of CLAD patients (Supplemental Figure 5A). Control lungs and HLNs as well as IPF HLNs showed upregulation of genes associated with tissue egress (*S1PR1* and *CCR7*). Surprisingly, CD8^+^ T cells from IPF HLNs showed the highest expression of genes associated with effector function (*IFNG* and *TNF*). Interrogation of the CLAD T cells identified that the upregulation of genes associated with cytotoxic potential were primarily derived from the most clonally expanded populations and those with a CD8^+^ TRM phenotype (Figure 4D and Supplemental Figure 5B). These cytotoxic genes were also found to be differentially upregulated within the subset of T cell clones that overlapped between the lungs and HLNs in CLAD (average log_2_[fold change] of 0.76, FDR-adjusted *P* value < 0.01) (Figure 4, E and F).

To determine whether protein content matched gene expression in these T cell populations, we performed intracellular staining for effector cytokines and cytolytic enzymes following in vitro stimulation with phorbol myristate acetate (PMA) and ionomycin. Intracellular expression of granzymes A and B was increased in CD8^+^ T cells from CLAD lungs compared with both IPF and control (Figure 4G and Supplemental Figure 6), although this increase was not statistically significant. This finding was also observed for granzyme B, but not granzyme A, in CD8^+^ T cells from CLAD HLNs. Granzyme A and B levels were also found to be elevated within the TRM subset of CD8^+^ T cells in CLAD lungs compared with control and IPF lungs (Supplemental Figure 5C). Interrogation of coexpression of cytotoxic effector proteins demonstrated increased coexpression of granzymes A, B, and K in CLAD lungs, but not in HLNs (Supplemental Figure 7). Interestingly, there was a significant shift in the ratio of granzyme B to granzyme K production between CD8^+^ T cells obtained from CLAD and IPF lungs, with CLAD CD8^+^ T cells producing proportionally more granzyme B and IPF lungs producing a relatively higher proportion of granzyme K (Figure 4H).

**Figure 5:**
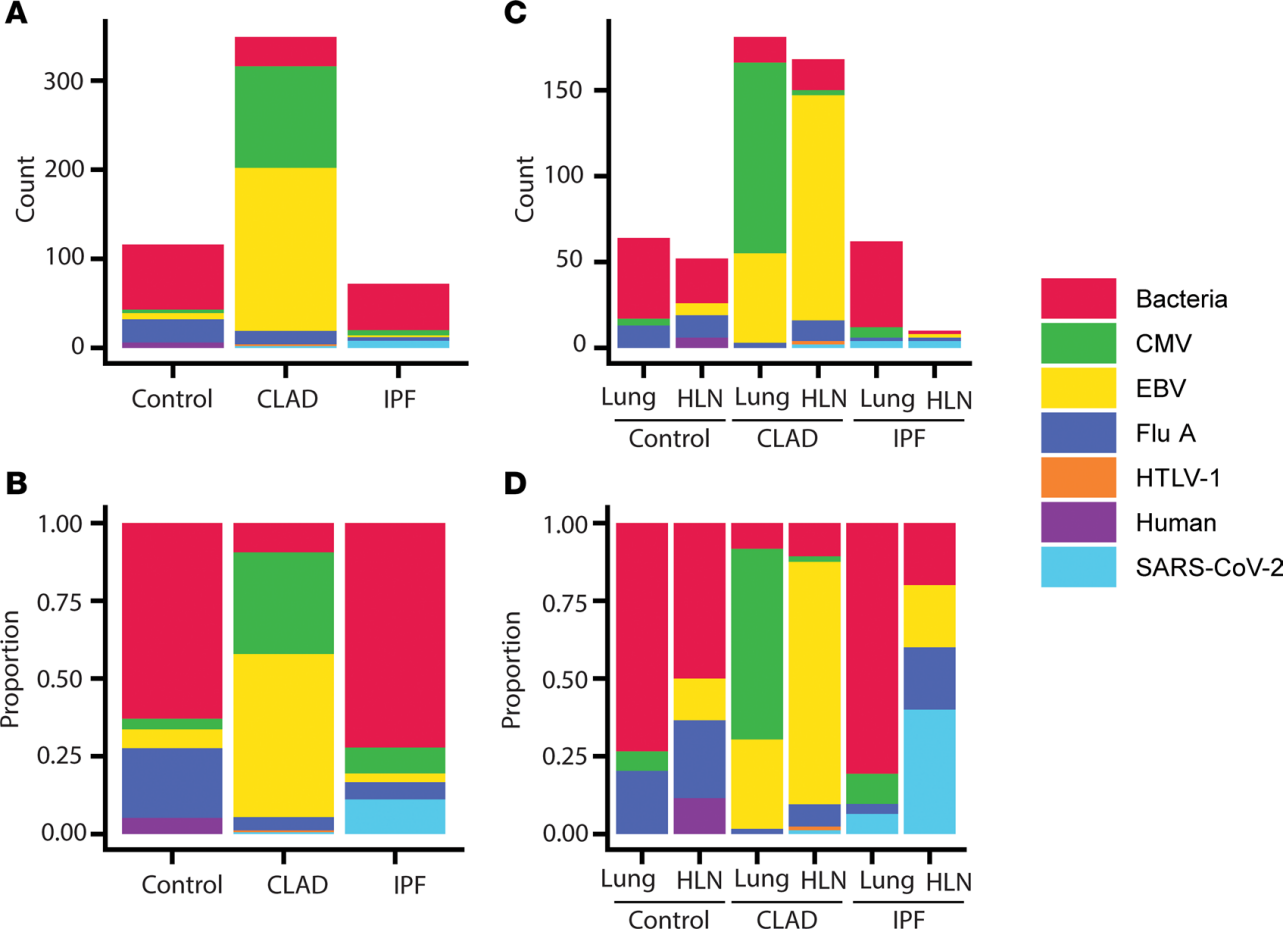
CLAD T cell repertoire is enriched for antigens associated with CMV and EBV. A machine-learning platform was used to estimate TCR epitope specificity from single-cell TCR sequencing data from single-cell suspensions of human lungs and HLNs with CLAD, IPF, and no underlying disease (Control). TCR epitope species specificity by disease — (**A**) total counts and (**B**) proportion of captured repertoire identified an increased number and proportion of EBV- and CMV-specific T cells. TCR epitope species specificity by disease and anatomic location — (**C**) total counts and (**D**) proportion of captured repertoire found CMV- and EBV-specific T cells in CLAD lungs, but a higher proportion of EBV-specific T cells in the CLAD HLNs.

Without cryopreserved donor tissue, we were unable to perform mixed lymphocyte reactions (MLRs) to determine the alloreactive potential of these clonally expanded, cytotoxic CD8^+^ TRMs. Instead, we used a machine-learning platform (ImmuneWatch Detect) with a curated IMWdb TCR epitope database to estimate TCR epitope specificity among T cells found in the lungs and HLNs from CLAD, IPF, and nondiseased lungs. Of the TCRs that were mapped to suspected epitopes, cytomegalovirus (CMV) and Epstein-Barr virus (EBV) epitopes comprised 85% of the CLAD repertoire, in stark contrast with control and IPF repertoires in which bacterial epitopes predominated and CMV and EBV represented a combined 9% and 11% respectively (Figure 5, A and B). CMV specificity came almost entirely from CLAD lung T cells. CLAD EBV specificity was found primarily among HLN T cells; however, 29% of the CLAD lung repertoire also demonstrated EBV specificity (Figure 5, C and D). These results suggest that much of the TRM accumulation in the lungs and HLNs of CLAD patients is related to herpesvirus responses. Previous work has shown that EBV-specific T cells can cross-react with the graft, leading to an alloreactive response (9). It remains unclear whether this population is specific to herpesviruses and/or contributing to a heterologous response (10, 11).

### Airway-centric cytotoxic NKG2D^+^CD8^+^ TRM accumulation is due to CLAD, not transplantation alone.

**Figure 6:**
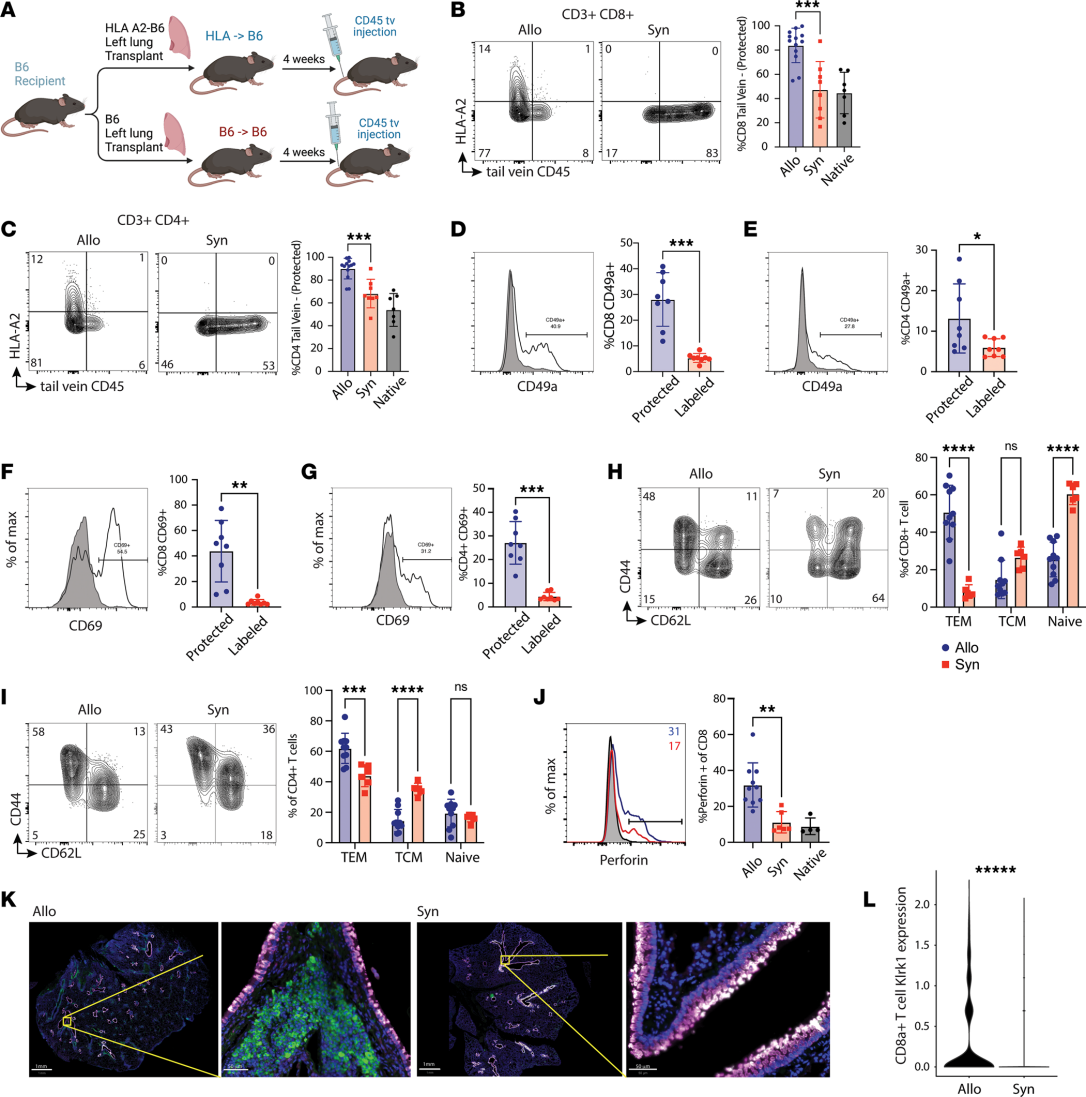
A murine model of orthotopic lung transplantation recapitulates enhanced cytotoxicity observed in human CLAD. (**A**) Schematic of murine orthotopic single-antigen mismatch lung transplant model. (**B** and **C**) Effect of lung transplantation on distribution of CD45^+^CD8^+^ T cells (**B**) and CD45^+^CD4^+^ T cells (**C**) in the lung. (**D** and **E**) The expression of CD49a on CD8^+^ T cells (**D**) and CD4^+^ T cells (**E**). (**F** and **G**) The expression of CD69 on CD8^+^ T cells (**F**) and CD4^+^ T cells (**G**) that were protected from intravenous CD45 labeling. (**H** and **I**) Memory T cell subsets were evaluated via expression of CD44 and CD62L on CD8^+^ (**H**) and CD4^+^ (**I**) T cells. (**J**) Perforin production from CD8^+^ T cells following transplantation. (**K**) Expression of NKG2D-encoding gene *Klrk1* in CD8^+^ T cells. Scale bars: 1 mm (low magnification) and 50 μm (high magnification). (**L**) Immunofluorescent staining for CD8^+^ T cells adjacent to mouse lung airways from Allo vs. Syn (green = CD8A, purple = CC10). *P < 0.05, **P < 0.01, ***P < 0.001, ****P < 0.0001 by Wilcoxon’s rank-sum test. Native, remaining native right lung.

The results of our translational studies showed that CLAD was associated with the accumulation of airway-centered, cytotoxic NKG2D^+^CD8^+^ TRMs. However, our control was nondiseased lungs from brain-dead donors who had not undergone transplantation; a far better control, lungs from transplant recipients without underlying CLAD, was unavailable, as these patients do not require re-transplantation. To determine whether our findings were due to CLAD and not merely a consequence of transplantation alone, we turned to a murine model of chronic rejection. Using a previously reported murine model of CLAD, C57BL/6 mice underwent single lung transplantation; donor lungs came from mice with a single HLA antigen mismatch or from a genetically identical donor. This experimental setup yielded 3 sets of lungs for analysis — the allogeneic transplanted lung (“Allo”), the syngeneic transplanted lung (“Syn”), or the remaining native lung (“Native”). 4 weeks after transplantation, lungs were harvested for analysis. Prior to sacrifice, all mice were intravenously injected with a fluorophore-conjugated anti-CD45 antibody to discriminate circulating T cells from lung TRMs (Figure 6A). We found a significant increase in the proportion of CD8^+^ TRMs (Figure 6B) and CD4^+^ TRMs (Figure 6C) within the Allo lungs compared with both Syn and Native lungs (*P* < 0.01). Congruent with these findings, analysis of the anti-CD45 circulating antibody–negative T cells (i.e., protected) confirmed that this subset did express a higher proportion of the canonical markers of tissue residency, CD49a and CD69 (Figure 6, D–G).

Major phenotypes of T cells can be grouped based on their antigen experience and localization using the 2 cell surface markers CD44 and CD62L, with CD44^+^CD62L^–^ cells representing TEM cells, CD44^+^CD62L^+^ representing TCMs, and CD44^–^CD62L^+^ as naive T cells. In keeping with our finding that most T cells in the Allo lungs had a TRM phenotype based on protection from circulating antibodies and upregulation of TRM markers, we found that most CD8^+^ T cells found in the Allo lung were TEMs, whereas most found in the Syn lungs were naive (Figure 6H). Similar to CD8^+^ T cells, most CD4^+^ T cells found in Allo lungs were TEMs (Figure 6I). However, in contrast with CD8^+^ T cells, most CD4^+^ T cells found in the Syn lungs were TCMs. At baseline, without stimulation, CD8^+^ T cells obtained from Allo lungs had a significantly increased ambient content of perforin (*P* < 0.01), suggesting a population of TRMs that have high cytotoxic potential (Figure 6J). Immunofluorescent imaging of formalin-fixed, paraffin-embedded Allo and Syn lungs at 4 weeks after transplantation demonstrated diffuse and robust accumulation of CD8^+^ T cells adjacent to the airways in Allo mice, as opposed to almost no CD8^+^ T cell accumulation in the Syn airways (Figure 6K).

**Figure 7:**
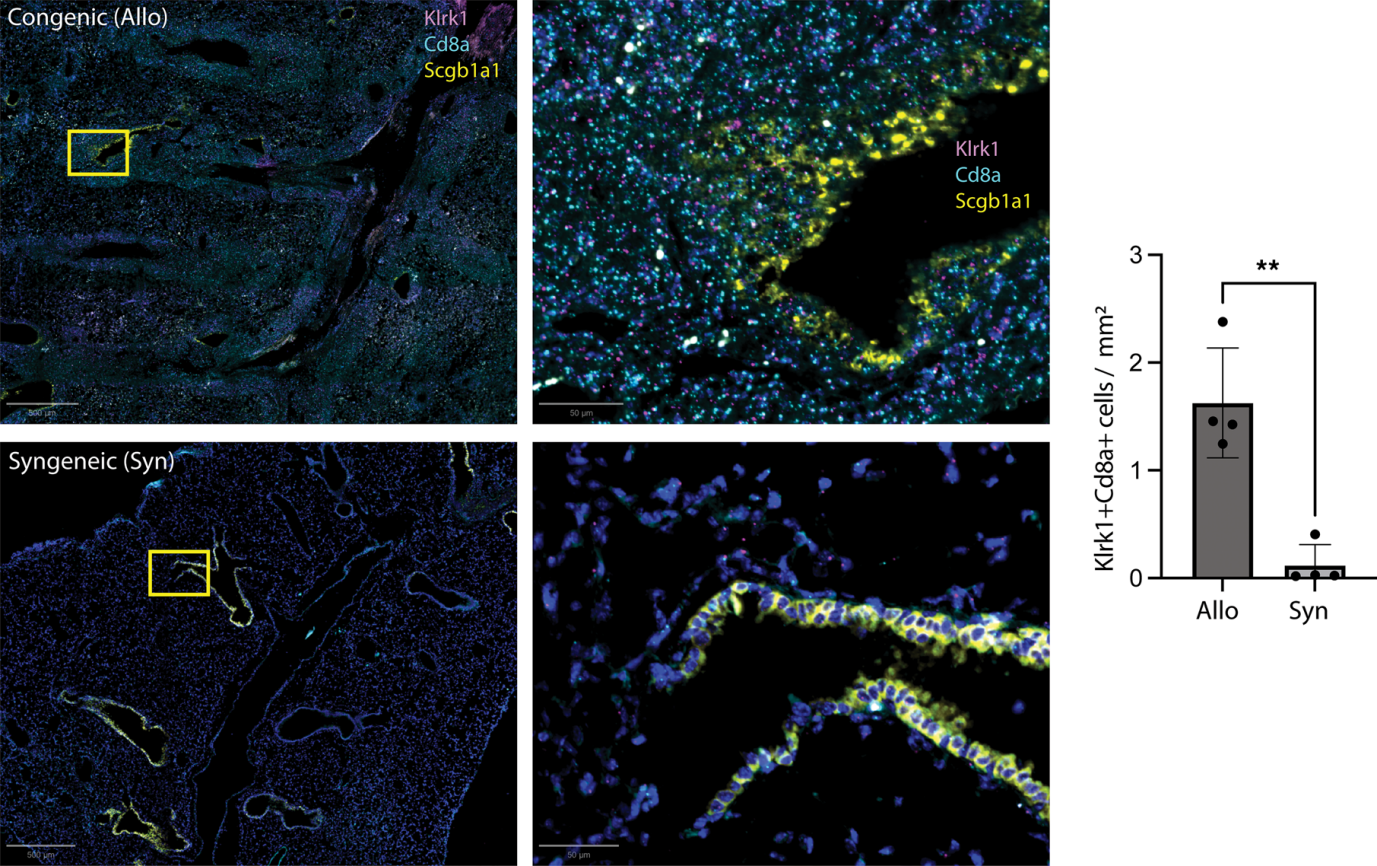
Accumulation of airway-centered *Klrk1*^+^ CD8^+^ cells in murine chronic rejection. Multispectral fluorescent RNA in situ hybridization of formalin-fixed, paraffin-embedded murine left-lung grafts with probes for *Klrk1*, *Cd8a*, and *Scgb1a1*. Samples were obtained at 4 weeks from either HLA→B6 (congenic) or B6→B6 (syngeneic) grafts. Scale bars: 500 μm (left) and 50 μm (right). Cumulative data presented on the right, with total *n* = 8 (4 congenic, 4 syngeneic). Values calculated as *Klrk1*^+^*Cd8a*^+^*Scgb1a1*^–^ cells per mm^2^ averaged across between 3 and 4 airways per sample. **P = 0.0015 by unpaired *t* test.

Using a publicly available single-cell RNA sequencing dataset from Allo and Syn lungs using this same murine HLA→B6 model, we compared the expression of *Klrk1* in a subset of CD8a^hi^ cells (12). We found an increased expression of *Klrk1* among CD8^+^ T cells from Allo mice (average log_2_[fold change] of 1.5, *P* < 1 × 10^–40^) (Figure 6L). Using multispectral fluorescent RNA in situ hybridization (RNAscope, Advanced Cell Diagnostics) we next tested the quantity and localization of *Klrk1-*expressing CD8^+^ T cells in formalin-fixed, paraffin-embedded congenic (Allo) and Syn murine lung allografts. In the congenic (Allo) grafts, we found the accumulation of large quantities of *Klrk1*^+^*Cd8a*^+^*Scgb1a1*^–^ cells adjacent to *Scgb1a1*^+^ epithelial cells (Figure 7). The quantity of airway-centered *Klrk1*^+^*Cd8a*^+^*Scgb1a1*^–^ cells was significantly greater in the congenic (Allo) compared with Syn grafts. This accumulation of *Klrk1*^+^*Cd8a*^+^ cells subjectively appeared to be associated with disruption of the normal columnar appearance of *Scgb1a1*^+^ epithelial cells. These findings support the notion that the accumulation of airway-centered, cytotoxic CD8^+^ TRMs with high *Klrk1* expression is due to chronic rejection and not an effect from transplantation alone.

**Figure 8:**
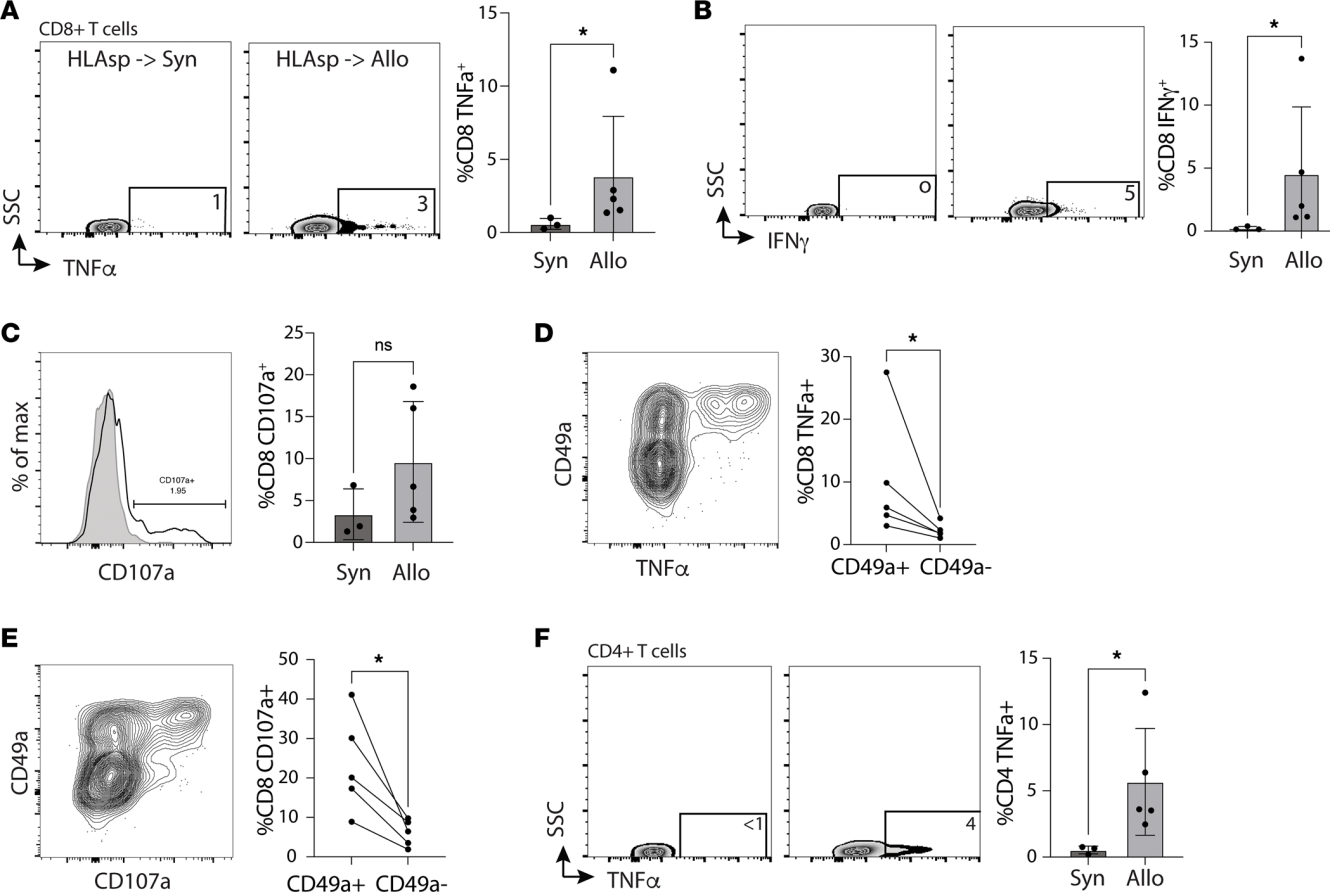
Mixed lymphocyte reaction (MLR) of syngeneic (Syn) or congenic (Allo) lungs with donor splenocytes. (**A**–**C**) Representative flow cytometry plot of CD8^+^ T cell production and cumulative data of (**A**) TNF-α and (**B**) IFN-γ expression after 15 hours of MLR in the presence of brefeldin A, and (**C**) CD107a cell surface exposure (surrogate marker of cytotoxic granule degranulation). For **A**–**C**, *n* = 5 (Allo), *n* = 3 (Syn). (**D** and **E**) Representative flow cytometry plot (left) of a surrogate marker of TRM (CD49a) versus (**D**) TNF-α production after 15-hour MLR and (**E**) CD107a cell surface exposure versus CD49a expression among Allo (*n* = 5) lungs. (**F**) Representative flow cytometry plot (left) of CD4^+^ T cell expression of TNF-α after 15-hour MLR and (right) cumulative data comparing Allo (*n* = 5) with Syn (*n* = 3) lungs. *P < 0.05 by unpaired *t* test. NS, no statistically significant difference.

To assess the alloreactive potential of CD8^+^ T cells found in the Allo lung, we performed an MLR with irradiated donor splenocytes from either an HLA mouse or C57BL/B6 mouse. After 15 hours of an MLR in the presence of brefeldin A, we found significantly increased production of TNF-α (Figure 8A and Supplemental Figure 8) and IFN-γ (Figure 8B) in CD8^+^ T cells from Allo lungs compared with Syn lungs, but no difference in a surrogate marker of degranulation (CD107a) (Figure 8C). Interestingly, most TNF-α production and degranulation (CD107a^+^) was from phenotypic TRMs based on CD49a expression (Figure 8, D and E; *P* < 0.05). We similarly found an increased production of TNF-α among CD4^+^ T cells found in Allo lungs compared with Syn lungs (Figure 8F), but there was not sufficient CD49a expression to test for differences in TRM versus non-TRM. These findings support the notion that the majority of the alloreactive response at 4 weeks is being driven by CD8^+^ TRMs. However, it is surprising that most T cells found within the Allo lungs did not have a positive response to donor antigen. There are a few potential explanations for a seemingly paradoxical finding of airway T cell accumulation but relatively low percentage of TNF-α^+^, IFN-γ^+^, and CD107a^+^ cells after MLR. It is possible that many of these T cells are accumulating via bystander activation (cytokine alone stimulation without the TCR engaging cognate antigen presented by MHC) and are not directly alloreactive. Alternatively, these are alloreactive cells that have become hypofunctional (exhausted) following antigen persistence. Further study is needed to explain these findings.

### NKG2D blockade attenuates pathophysiologic changes of CLAD in murine model.

**Figure 9:**
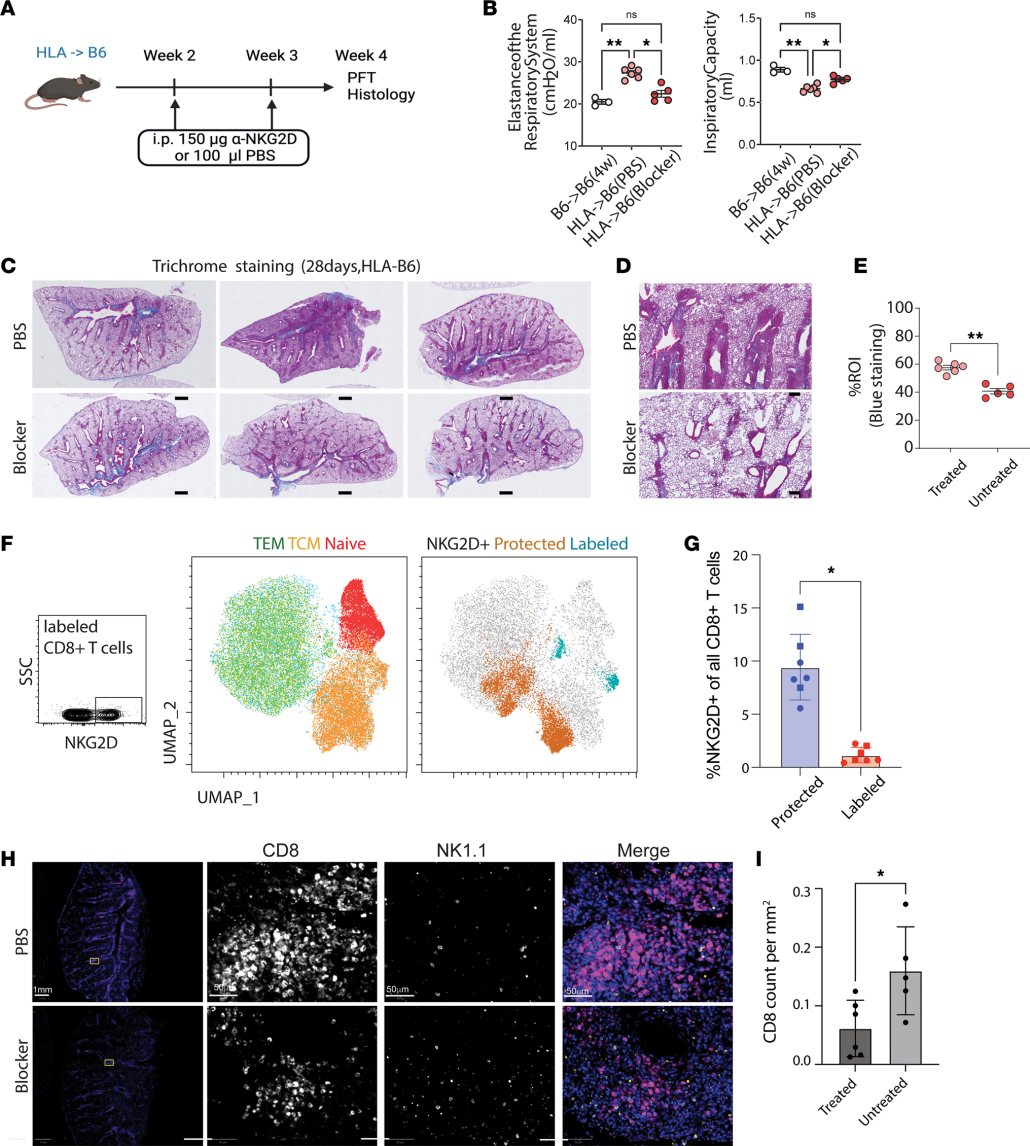
NKG2D blockade attenuates pathophysiologic changes in murine model of CLAD. (**A**) Experimental approach. (**B**) Respiratory system elastance and inspiratory capacity after syngeneic single lung transplant (*n* = 3), allogeneic single lung transplant (*n* = 5), and allogeneic single lung transplant with NKG2D blockade (*n* = 6). (**C** and **D**) Trichrome staining of connective tissue deposition in mouse allogeneic lung transplants with and without NKG2D blockade at (**C**) ×4 and (**D**) ×10 magnification. (**E**) Cumulative data quantifying trichrome staining. (**F**) Representative flow cytometry plot showing gating strategy for NKG2D positivity (left) and UMAP of concatenated CD8^+^ T cells obtained from murine allografts showing major T cell phenotypes, including TEM (CD44^+^CD62L^–^), TCM (CD44^+^CD62L^+^), and naive cells (CD44^–^CD62L^–^) (middle), and UMAP highlighting the NKG2D^+^ cells in the protected (orange) and labeled (blue) compartments (includes cells from *n* = 7 allografts, 3 from PBS and 4 from NKG2D blocker, downsampled to have equal numbers of cells from each lung). (**G**) Cumulative data comparing percentage of all CD8^+^ T cells that are NKG2D^+^ within the protected versus labeled allografts; square symbols are those from PBS-treated and circles are those from NKG2D blocker–treated allografts. (**H**) Immunofluorescence imaging for CD8 (purple) and NK1.1 (yellow) in mouse allogeneic lung transplants with and without NKG2D blockade (first column at ×4, remaining at ×40). (**I**) CD8^+^ T cell concentration in mouse allogeneic lung transplants with (Treated) and without (Untreated) NKG2D blockade. *P < 0.05; **P < 0.01 by unpaired *t* test.

As described above, C57BL/6 mice underwent allogeneic or syngeneic left lung transplantation. At weeks 2 and 3 after transplantation, mice were injected intraperitoneally with either an NKG2D-blocking antibody (Bio X Cell, clone CX5) or phosphate-buffered saline (PBS) (Figure 9A). At 4 weeks after transplantation, untreated Allo mice demonstrated increased respiratory system elastance (decreased compliance) compared with Syn mice (Figure 9B). In line with this finding, decreased inspiratory capacity was also observed in the Allo mice (Figure 9B). Significant reversal of pathophysiology (decreases in elastance and increases in inspiratory capacity) were observed in Allo mice that underwent treatment with systemic NKG2D blockade (Figure 9, A and B). Peribronchiolar fibrosis, identified via connective tissue deposition on trichrome staining, was also reduced following NKG2D blockade (Figure 9, C–E). Using flow cytometry of single-cell suspensions obtained from allografts, we found that all NKG2D^+^CD8^+^ T cells were of a memory phenotype (either TEM or TCM) (Figure 9F). NKG2D^+^CD8^+^ T cells were predominantly TRMs; however, there was no difference in the proportion of NKG2D^+^CD8^+^ T cells based on whether they had received systemic NKG2D blockade or PBS (Figure 9G). Immunofluorescence images of mouse lungs with CD8 staining and NK1.1 staining were quantitatively analyzed (Figure 9H). Allo mice demonstrated an increased lung CD8^+^ T cell infiltration; this was significantly reduced following treatment with NKG2D blockade (Figure 9, H and I). NK cells were affected to a limited, nonsignificant degree (Supplemental Figure 9). These results show that systemic NKG2D blockade results in the diminished accumulation of airway-centered CD8^+^ TRMs, but does not alter the proportion of NKG2D^+^ cells.

## Discussion

Following lung transplantation in humans, recipient-derived T cells are recruited to the allograft, presumably in the setting of rejection or infection, and take up long-term residency as noncirculating TRMs, predominantly in or around the airways (6). The migration of T cells from the perivascular space to the airways after acute cellular rejection and the finding of CD8^+^ T cell–mediated killing of airway basal cells and club cells suggests that these airway-centered T cells have, in at least some capacity, alloreactive activity (5, 7, 13). Here, we explore the phenotype, location, and function of TRMs obtained from human CLAD lungs and HLNs as well as in a murine model of CLAD, and identify a potential therapeutic target (NKG2D) to prevent the accumulation of airway-centered, cytotoxic CD8^+^ TRMs. Using combined single-cell RNA and TCR sequencing, we found that CLAD lungs and HLNs showed a unique clonal expansion of recipient-derived, cytotoxic CD8^+^ T cells that were phenotypic TRMs. These cytotoxic TRMs upregulated the cell surface protein NKG2D, commonly found on both NK cells and cytotoxic CD8^+^ T cells. Importantly, many of these clonally expanded CD8^+^ T cells were estimated to be specific for herpesviruses (CMV and EBV) and had a high degree of clonal overlap between the CLAD lungs and CLAD HLNs, far greater than that in either control or IPF lungs and HLNs. Using a single antigen mismatch murine model (HLA→B6), we confirmed that the accumulation of cytotoxic CD8^+^ TRMs in the allograft was due to chronic rejection and not a by-product of the surgical process of transplantation. Finally, we show that the systemic administration of NKG2D blockade led to the partial reversal of CLAD airway remodeling and a diminished number of airway-centered CD8^+^ T cells. These findings suggest that NKG2D is a potential target for alloreactive, cytotoxic CD8^+^ T cells in the setting of CLAD.

It has long been known that secondary lymphoid organs (SLOs) are not required for the initiation of solid organ allograft rejection, a finding confirmed in murine models of lung transplantation (14, 15). In experimental models, initial priming of alloreactive T cells occurred within the lung allograft, a finding consistent with the ability of tertiary lymphoid organs to contribute to rejection by generating alloreactive memory T cell populations (15, 16). However, SLOs may be critical for sustained alloresponses leading to chronic airway inflammation (17). In this study, we found that recipient-derived lymphocytes infiltrated donor-derived lymph nodes found at the tertiary carina that were transplanted along with the lung and replaced all donor-derived immune cells. Importantly, the recipient-derived CD8^+^ T cells found in these “hijacked” lymph nodes showed a 100-fold increase in clonal overlap with lung-derived CD8^+^ T cells when compared with either control or IPF lungs. These finding support prior mouse work suggesting initial priming of alloreactive T cells in the lung and nontransplant work showing the lung draining lymph node to be a reservoir of influenza-specific effector cells in an experimental model of influenza infection (15, 18). We propose that the HLN is acting as a reservoir of alloreactive T cells working to sustain a chronic alloresponse. However, definitive evidence of their alloreactive potential is lacking and future investigation will focus on both the direct alloreactive potential of lung TRMs as well as the possible role of heterologous immune responses in the development of CLAD.

Heterologous immunity, or the ability of a pathogen-specific T cell to recognize diverse epitopes, including alloantigen, may explain the association of CMV pneumonitis with the development of CLAD after lung transplantation (10, 19, 20). TCR cross-reactivity in most contexts can be beneficial, enabling an adaptive immune response to a multitude of existing and emerging pathogens, the quantity of which outnumber the estimated pool of available TCRs (21). However, in the context of transplantation, this can lead to common viral pathogens contributing to alloimmunity, a process previously reported for both CMV- and EBV-specific T cells. Following kidney transplantation, CMV-specific T cells found in both the circulation and allograft elicit alloreactive responses (11). EBV-specific T cells have similarly been shown to be cross-reactive with alloantigens in humans (9). We found that clonally expanded, cytotoxic, CD8^+^ TRMs in both the lung allograft and HLNs were enriched for EBV- and CMV-specific epitopes using a machine-learning platform to estimate TCR specificity. It remains unknown whether these T cells are cross-reactive with the allograft, but their abundance at the time of CLAD supports future study into this potential mechanism of injury.

NKG2D has been traditionally considered to activate production of cytotoxic effector molecules in NK cells upon binding of its cognate ligands MHC class I polypeptide–related sequence A (MICA) and MICB (22). Its role in CD8^+^ T cells in CLAD is less well established. Previous work has highlighted the important role of NKG2D on NK cells in primary graft dysfunction immediately following lung transplantation (22–24). Increased expression of NKG2D’s cognate ligand, MICB, has also been observed in the bronchoalveolar lavage of LTRs with primary graft dysfunction and has been shown to correlate with lung function outcomes as far out as 1 year after transplantation (24). In vitro experiments demonstrated that NKG2D blockade abrogated NK cell–mediated killing of lung epithelial cells (24). Our study clearly demonstrates that systemic NKG2D blockade diminishes both airway remodeling after transplantation and the accumulation of cytotoxic CD8^+^ TRMs, but provides no insight into the impact on established TRM function. There are many possible ways that NKG2D blockade may have contributed to both reduced airway remodeling and diminished CD8^+^ TRM accumulation. *Klrk1*, the gene encoding NKG2D, is one of a few genes that is increasingly upregulated in T cells after repeated antigen exposure (25). It is possible that blocking NKG2D prevents either the formation or persistence of airway CD8^+^ TRMs, a process that could be driven by continuous antigen exposure. Similarly, systemic NKG2D blockade may have impacted CD8^+^ TRM accumulation and airway remodeling indirectly via its impact on NK cells. Although we cannot rule out this possibility, we suspect this is unlikely, considering NKG2D inhibition was introduced 2 weeks after transplantation, a time point when T cell activity would be expected to predominate over NK cell activity and a preserved quantity of lung NK cells between conditions (26).

Our work provides further evidence of the importance of CD8^+^ T cell interactions in the development of CLAD. Type 1–associated gene expression signatures have previously been observed in the distal airways of LTRs with CLAD compared with those without CLAD (27). Khatri et al. previously demonstrated increased cytotoxic CD8^+^ T cell infiltration in CLAD lungs, colocalizing with enhanced MHC-I expression on basal lung epithelial cells, findings that were mediated by IFN and JAK/STAT signaling (5). Consistent with our findings, they also observed increased gene expression of *GZMA*, *GZMB*, and *IFNG* within CD8^+^ T cells from CLAD lungs. Our data add to this body of evidence by demonstrating the elevated and simultaneous production of granzyme A and granzyme B by CD8^+^ T cells in the CLAD lung compared with control. While causal association cannot be established from our data, the mounting evidence strongly supports a pathogenic role for cytotoxic CD8^+^ T cells.

IPF lungs were notable for the relative accumulation of non–tissue-resident TEMs, with a shift toward a granzyme K–producing phenotype. Granzyme K is a serine protease with relatively poor cytotoxic capacity when compared with others like granzymes A and B (28). It is upregulated in senescent CD8^+^ T cells during normal aging in a process called inflammaging (29); the accumulation of granzyme K–producing CD8^+^ T cells has been described in chronically inflamed tissues in humans (30). Unlike other serine proteases, granzyme K has extracellular effects, including the induction of proinflammatory cytokine secretion by fibroblasts by activating the cell surface protein, protease-activated receptor 1 (31). Further study is required to determine the physiologic impact of granzyme K–producing memory CD8^+^ T cells, if any, on the development or progression of pulmonary fibrosis.

This study has limitations. The low rate of re-transplantation limited the number of explanted CLAD lungs available for analysis, a limitation we believe we address by adapting a murine OLT model of CLAD. Additionally, our human control samples, lungs from donors whose organs were rejected for clinical transplantation, were suboptimal for a variety of reasons: their lack of sustained immunosuppression and the potential immune changes from brain death (32). A more meaningful control would be lungs and HLNs from LTRs without CLAD, a source that is thankfully, not regularly available. We are reassured that our findings in CLAD would hold when compared to non-CLAD LTRs in light of the recent work by Khatri et al. who did have access to these precious samples (surgical lung biopsies from LTRs without CLAD, obtained for non-CLAD indications) (5). In their study, CLAD lungs, in contrast with lungs from LTRs without CLAD, demonstrated increased colocalization of CD8^+^ T cells and basal epithelial cells in CLAD in conjunction with ligand-receptor interactions between the 2 cell types and apoptosis of the basal cells in direct proximity to the CD8^+^ T cells. We could not definitively show the alloreactive potential of T cells found in human CLAD samples due to a lack of cryopreserved donor tissue. Finally, our murine model does have notable limitations. These include the absence of systemic immune modulators, and that our model is a single-antigen, xenotransplant model, which may impact the translation to human studies.

In conclusion, we show that CLAD lungs and HLNs are enriched for clonally expanded and overlapping cytotoxic CD8^+^ TRMs that express the co-receptor NKG2D, and that blocking NKG2D in vivo can attenuate airway remodeling after transplantation. Future work will focus on optimizing timing and means of delivery as well as delineating the impact of NKG2D blockade on NK cell versus cytotoxic CD8^+^ T cell responses and test how this may impact humoral immunity.

## Methods

### Sex as a biological variable.

We included nearly equal numbers of explanted lungs from men and women undergoing either transplantation or as organ donors. However, sex was not included in our analyses. We performed mouse OLT on male mice only due to technical constraints.

### Human sample processing.

See Supplemental Table 1 for tissue donor details. Following transplantation, lung and HLN tissues were cross sectioned and placed into a tissue cartridge in zinc formalin fixation solution for 48 to 72 hours. Sections were then placed in 70% ethanol prior to paraffin embedding. Samples were embedded within 1 week of transplantation. To process tissue for flow cytometry analysis, lungs were injected with digest media composed of RPMI with 10% FBS (Gibco) and 1% pen/strep, DNase (Sigma-Aldrich), and Collagenase P (MilliporeSigma). The tissue was then manually cut into fine pieces and transferred to a GentleMACS Dissociator (Miltenyi Biotec) for 30 minutes at 37°C. Following dissociation, tissue was filtered in the following order: first through a stainless-steel mesh filter, then through a 100-μm filter, and then through a 70-μm filter. The suspension was then centrifuged at 300*g* for 5 minutes. Cells were then counted and frozen at –80°C for future analyses.

### Flow cytometry and cytokine analysis.

For phenotypic analysis, single-cell suspensions of lung and HLN cells were surface stained (CD3, Zombie UV, HLA-DR, CD8, CD25, CD4, CD20, CD45RA, CD69, CD31, CCR7, donor/recipient HLA, CRTAM, NKG2D, CD45, GNLY). Antibody details can be found in Supplemental Table 2. Cells were fixed (BD Cytofix Fixation Buffer) and analyzed via flow cytometry on a Cytek Aurora spectral flow cytometer. For functional analysis, cells were simulated with ImmunoCult CD3/CD28 T cell activator (STEMCELL Technologies) at a concentration of 25 μL per mL of cell suspension. After 5 hours, cells were washed with PBS with 5% FBS and surface stained (CD3, Zombie UV, CD56, CD8, CD4, CD107a, PD1, CCR7, donor/recipient HLA, CCL5, CD103, NKG7, CD45). Cells were then fixed and permeabilized (BD Perm/Wash buffer) and intracellular cytokine staining was performed (granzyme B, granzyme A, granzyme K, perforin, IL-10, NKG7, TNF-α, and IFN-γ). Cells were run on an Aurora spectral flow cytometer (Cytek Biosciences) and analyzed using FlowJo analysis software (Tree Star).

For in vitro analysis of cytokine production following incubation with MICA with and without NKG2D blockade, 200,000 cells from whole-lung homogenate were cultured under the following conditions for 4 days: unstimulated CD3/CD28 (STEMCELL Technologies), stimulated CD3/CD28 plus MICA (Novus Biologicals), and stimulated CD3/CD28 plus 14 μL MICA plus 1 μL NKG2D block (Invitrogen). After 4 days, cell supernatants were collected for cytokine analysis via LEGENDPlex (Human CD8/NK Panel 13-Plex, BioLegend) according to the manufacturer’s instructions. Samples were processed undiluted and at 1:10. Samples were analyzed on a Cytek Aurora using Qognit software (BioLegend).

### Single-cell RNA/TCR sequencing.

Surgically explanted lungs and HLNs from 9 patients were obtained (3 LTRs with CLAD undergoing re-transplantation, 3 healthy donor controls without chronic lung disease who were deemed not to be candidates for organ transplantation, and 3 patients with IPF undergoing lung transplantation). Individual samples were tagged with unique hashtag oligonucleotides (BioLegend). Recipient-derived T cells from each of these 18 samples (9 patients, lungs and HLNs from each) were isolated via FACS for live singlets that were CD45^+^Lineage^–^ (where Lineage^–^ is CD20^–^CD19^–^CD56^–^CD14^–^) CD4^+^ and/or CD8^+^ pan-HLA^+^ recipient–specific HLA^+^, as previously described (7). Sorted cells were captured for single-cell RNA and TCR sequencing using the 10× Genomics 5′ assay. Libraries were sequenced using the Illumina platform. Alignment, filtering, barcode counting, and unique molecular identifier counting were performed using 10× Genomics CellRanger v5 and CellRanger VDJ pipelines.

### Single-cell RNA/TCR data processing and analysis.

Downstream analysis was performed in R using Seurat v3 and scRepertoire packages (33, 34). Normalization and variance stabilization of count data were performed using scTransform (35). Individual Seurat objects were integrated using Seurat’s canonical correlation analysis with 3,000 integration anchors based on the previously transformed normalization values. A small number of contaminating cells of myeloid descent were removed from analyses based on CD68 expression within distinct clusters (36). T cells were retained for downstream analysis if they contained an amino acid sequence annotation for the CDR3 β chain. TCR epitope specificity analysis was performed using the ImmuneWatch DETECT platform and IMWdb epitope database with a binding score cutoff of 0.2 or greater (v1.0, https://www.immunewatch.com/detect). All code used for single-cell analyses can be found in the following GitHub repository, including a list of all R packages used for analyses: https://github.com/kmoghbeli/CLAD-NKG2D

### Immunofluorescence imaging.

Paraffin-embedded lung sections were first deparaffinized by submerging in xylene for 5 minutes twice, and then in 100% ethanol for 5 minutes twice. The slide holder was then submerged in 95% ethanol for 5 minutes twice, followed by 70% ethanol twice, and then was submerged in deionized water. For antigen retrieval, slides were steamed with Dako solution (Agilent) in a decloaking chamber for 17 minutes at 95°C. For surface staining, slides were washed twice with PBS for 10 minutes; for intracellular staining, the slides were permeabilized once for 10 minutes with 0.4% Triton X-100. Slides were then blocked for 1 hour at room temperature. For surface staining, antibodies were diluted in a 1:100 mixture of blocking buffer and PBS; for intracellular staining, antibodies were diluted in a 1:100 mixture of blocking buffer diluted in PBS plus Triton X-100. After overnight staining, slides were washed with PBS or PBS plus Triton X-100. In cases where secondary antibody was required, staining occurred at a dilution of 1:400 and slides were incubated for 1 hour at room temperature. For DAPI staining, slides were washed with PBS 3 times for 5 minutes each. Slides were stained with 1 μL DAPI intermediately in 1 mL PBS for 3 minutes at room temperature, and then the slides were again washed 3 times for 5 minutes each. Slides were then dried, mounted, and covered with Diamond Antifade solution (Invitrogen) and the slides were secured using a clear coat of nail polish. Stained slides were stored at 4°C until imaging. For CD8^+^ T cell quantitation, QuPath Open Source BioImaging Analysis (https://qupath.github.io/) was used to generate a tiled figure; total numbers of nucleated cells and total numbers of CD4^+^ and CD8^+^ cells per 500 mm^2^ were quantified.

### Multispectral fluorescent RNA in-situ hybridization (RNAscope).

Mouse lung grafts were fixed in 10% formalin and then paraffin embedded. Sections (5 μm) were first deparaffinized in xylene for 5 minutes (twice), dehydrated in 100% ethanol for 2 minutes (twice), and then left to dry. Hydrogen peroxide was then added for 10 minutes at room temperature and slides were washed with MiliQ distilled water (twice). Antigen retrieval was performed with RNAscope Target Retrieval Reagents (Advanced Cell Diagnostics) for 15 minutes at 110°C. Probes for *Klrk1*, *Cd8a*, and *Scgb1a1* were purchased from Advanced Cellular Diagnostics. Slides were prepared per the manufacturer’s specifications. Images were captured with an Olympus BX43 microscope with slide scanner and QuPath was used for imaging analysis. From each slide, annotations were made on 3–4 airways starting from the apical surface of the columnar epithelium and extending to a 100 mm depth of lung tissue. Nucleated cells were counted, intracellular detections for each channel was quantified, and the number of *Cd8a*^+^*Klrk1*^+^*Scgb1a1*^–^ cells were detected per unit area.

### Animal experiments.

Adult male C57BL/6 and C57BL/6-*Mcph1^Tg(HLA-A2.1)1Enge^*/J mice (The Jackson Laboratory) were utilized for OLT as previously described (37). For ex vivo and flow cytometry analysis, mice were injected via the tail vein with an anti-CD45 antibody to discriminate circulating versus resident T cells. For in vivo NKG2D blockade, HLA→B6 mice received an intraperitoneal injection of either 150 μg (17 μL) of anti-NKG2D (InVivoMAb anti–mouse NKG2D, clone CX5) in 100 μL of sterile PBS or 100 μL of PBS alone at week 2 and week 3 after OLT. Pulmonary function was evaluated as previously described (12).

### MLR.

To assess alloreactivity in our mouse lung allograft model, 1 × 10^6^ recipient lung cells were stained with 1 mL of CFSE (BioLegend) diluted 1:100 in PBS for 4 minutes at 37°C. Following incubation, cells were quenched with 2 mL of chilled FBS or human serum, and then washed twice with PBS. Stimulator cells from donor mice were irradiated at 30 Gy for 14 minutes. Recipient and donor cells were combined in a 1:1 ratio and incubated at 37°C for 5 days. Subsequent cytokine production and CFSE dilution were evaluated via flow cytometry as described above on a Cytek Aurora.

### Statistics.

All gene expression, flow cytometry proportion, and immunoassay results from human samples were compared using Wilcoxon’s rank sum test with results adjusted for false discovery using the Benjamini-Hochberg correction. For murine models, the 2-tailed, unpaired *t* test was used to test for statistical significance between cellular proportions between Allo, Syn, and Native lungs. The paired *t* test was used to compare within-mouse differences of labeled versus protected cells.

### Study approval.

Explanted lungs and HLNs from study participants undergoing re-transplantation were obtained after receiving approval from the University of Pittsburgh Institutional Review Board (IRB identification: STUDY21080113). Explanted lungs from research-consented organ donors whose lungs were rejected for clinical transplantation were obtained following written approval from our institutional Committee for Oversight of Research and Clinical Training Involving Decedents (CORID ID: 1116). Animal experiments were carried out in accordance with the University of Pittsburgh’s IACUC guidelines for the use and care of laboratory animals.

### Data availability.

Deidentified data will be made available upon written request to the corresponding author. All RNA sequencing data are publicly available via the NCBI Gene Expression Omnibus (GEO) under the accession number GSE285515. All code used to perform analyses are available via the following GitHub page: https://github.com/kmoghbeli/CLAD-NKG2D

## Author contributions

KM and MAL wrote the original draft of the manuscript, generated figures, and curated and analyzed data. The order of first authors was determined by the proportion of manuscript written. MB conducted experiments and analyzed data (murine studies). AC, ZIL, MA, and MR conducted experiments (human studies). BC conducted experiments (murine injections, tissue procurement). JS, KN, and DJK provided project administration, conducted experiments, and curated data (human tissue procurement). KC and LF conducted experiments (single-cell RNA cell capture and library preparation). TO generated figures and analyzed data (pathology). ZZ and XW conducted experiments (orthotopic lung transplants). JFM provided resources. OE conceptualized the study and developed methodology. MES conceptualized the study, developed methodology, acquired funding, and supervised the study. All authors reviewed and edited the manuscript.

## Supplementary Material

Supplemental data

Supporting data values

## Version 1 02/24/2025

Electronic publication
