# Supplementary material for: NKG2D blockade impairs tissue-resident memory T cell accumulation and reduces chronic lung allograft dysfunction
Source: JCI Insight. 2025 Feb 24;10(4):e184048. doi: 10.1172/jci.insight.184048 (PMC11949055; doi:10.1172/jci.insight.184048)
Supplement: Supplemental data [file jciinsight-10-184048-s091.pdf]

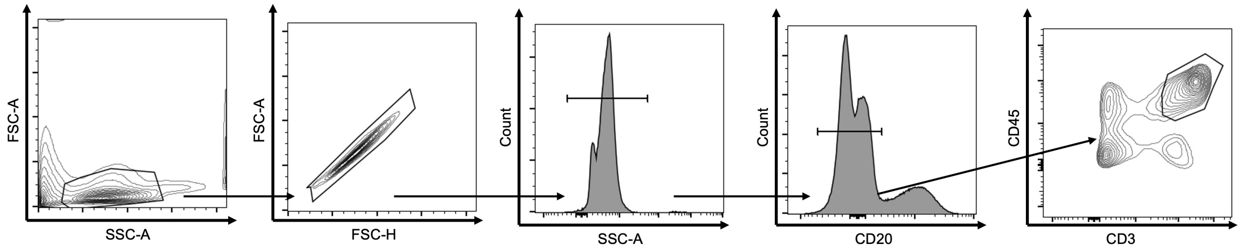

Figure S1. T cell flow identification via flow cytometry

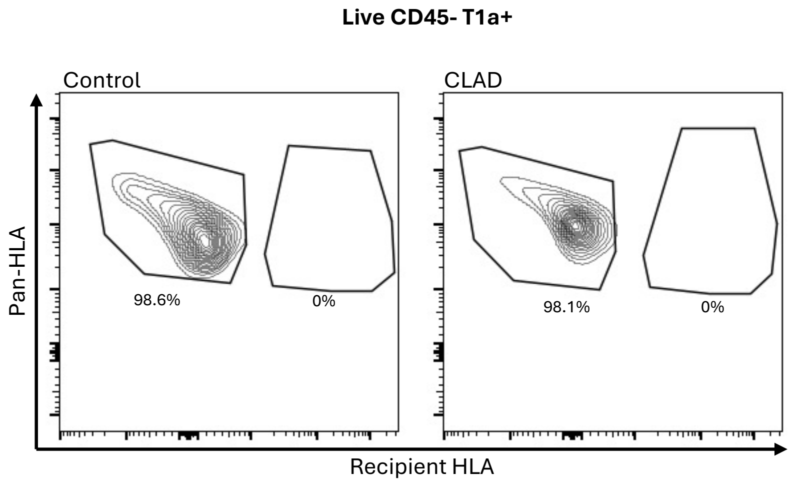

S2. Representative flow cytometry plot showing cellular origin (donor versus recipient based on human leukocyte antigen (HLA) discrepancies) of CD45-T1a+ cells in the lung draining lymph node. Cells were universally of donor origin (pan-HLA positive, recipient HLA negative).

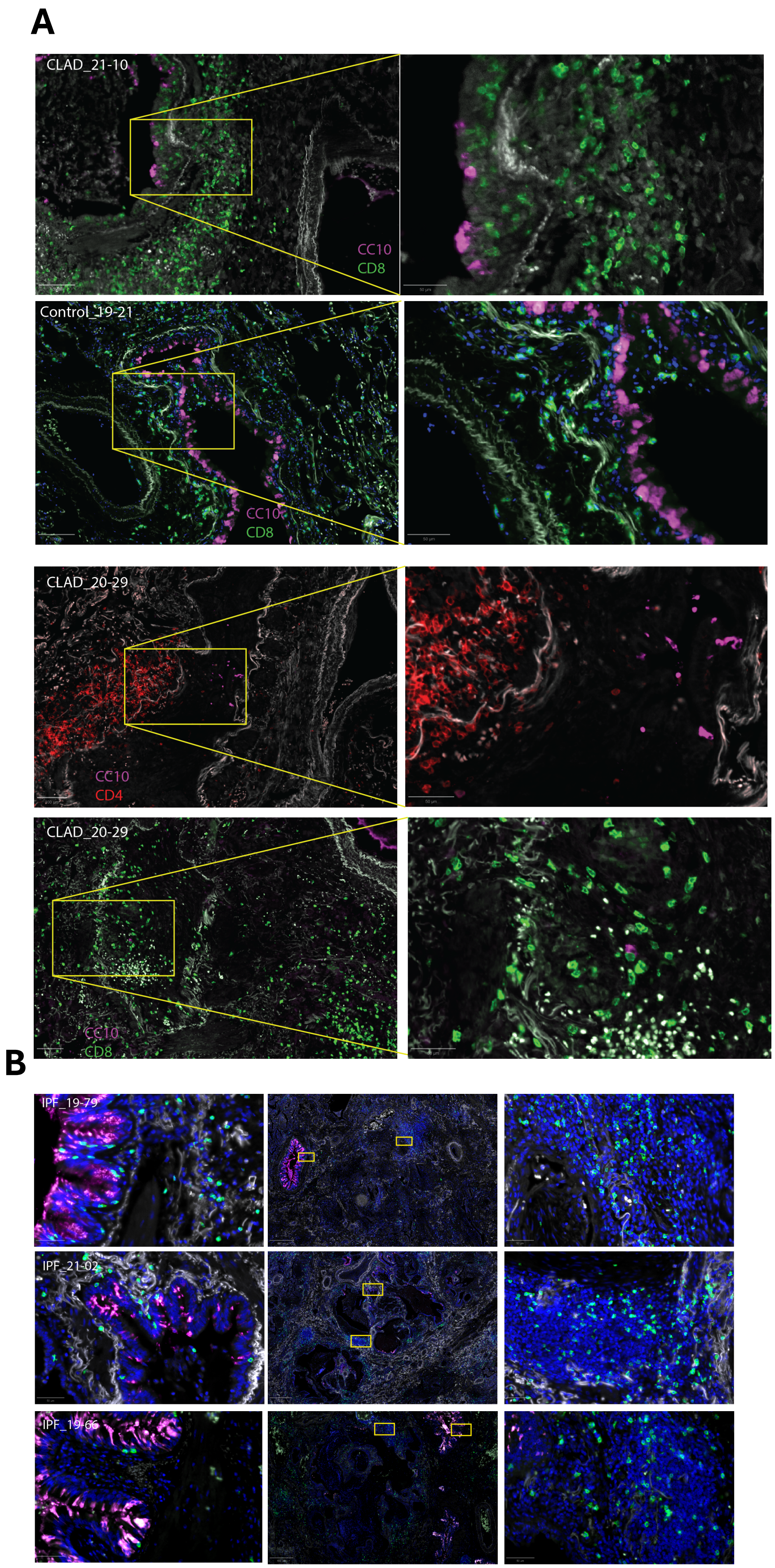
A
CLAD_21-10
CC10
CD8
Control_19-21
CC10
CD8
CLAD_20-29
CC10
CD4
CLAD_20-29
CC10
CD8
B
IPF_19-79
IPF_21-02
IPF_19-66

Figure S3: Lung CC10, CD8, and CD4 co-localization. A) CLAD and Control lung CC10 and CD8 co-localization (top 2 panels) and CC10 and CD4 co-localization (bottom 2 panels). B) IPF lung CC10, CD8, and CD4 co-localization. (Purple = CC10, Green = CD8, Red = CD4).

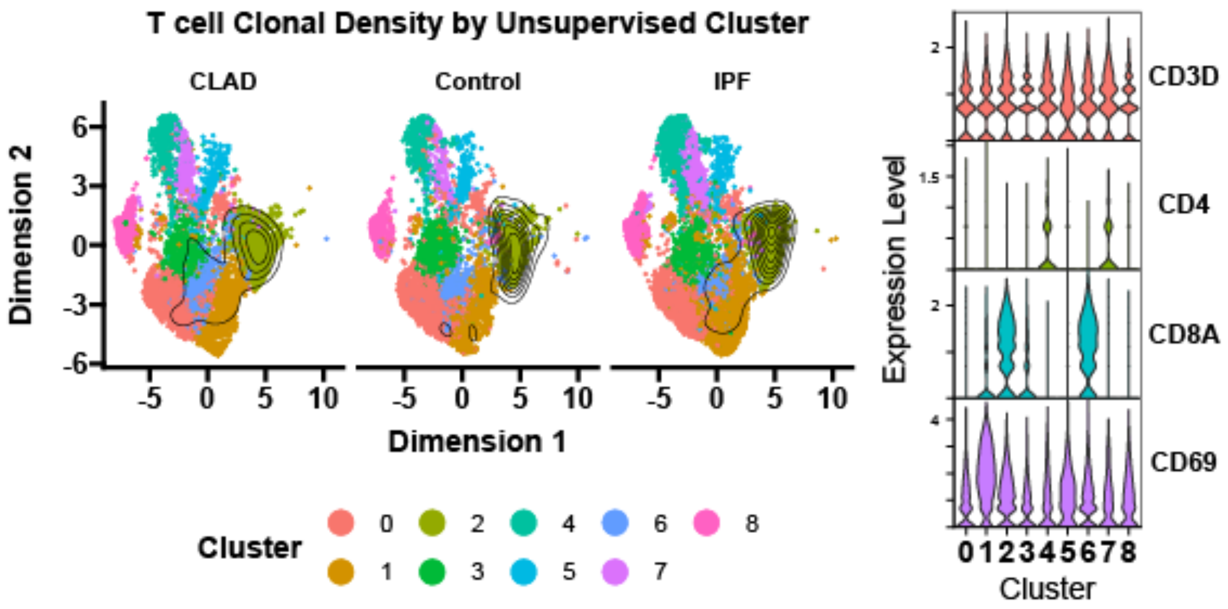

S4. Unsupervised clustering of T cells identifies a specific cluster (2) that contains a population of CD8A high cells that are clonally expanded.

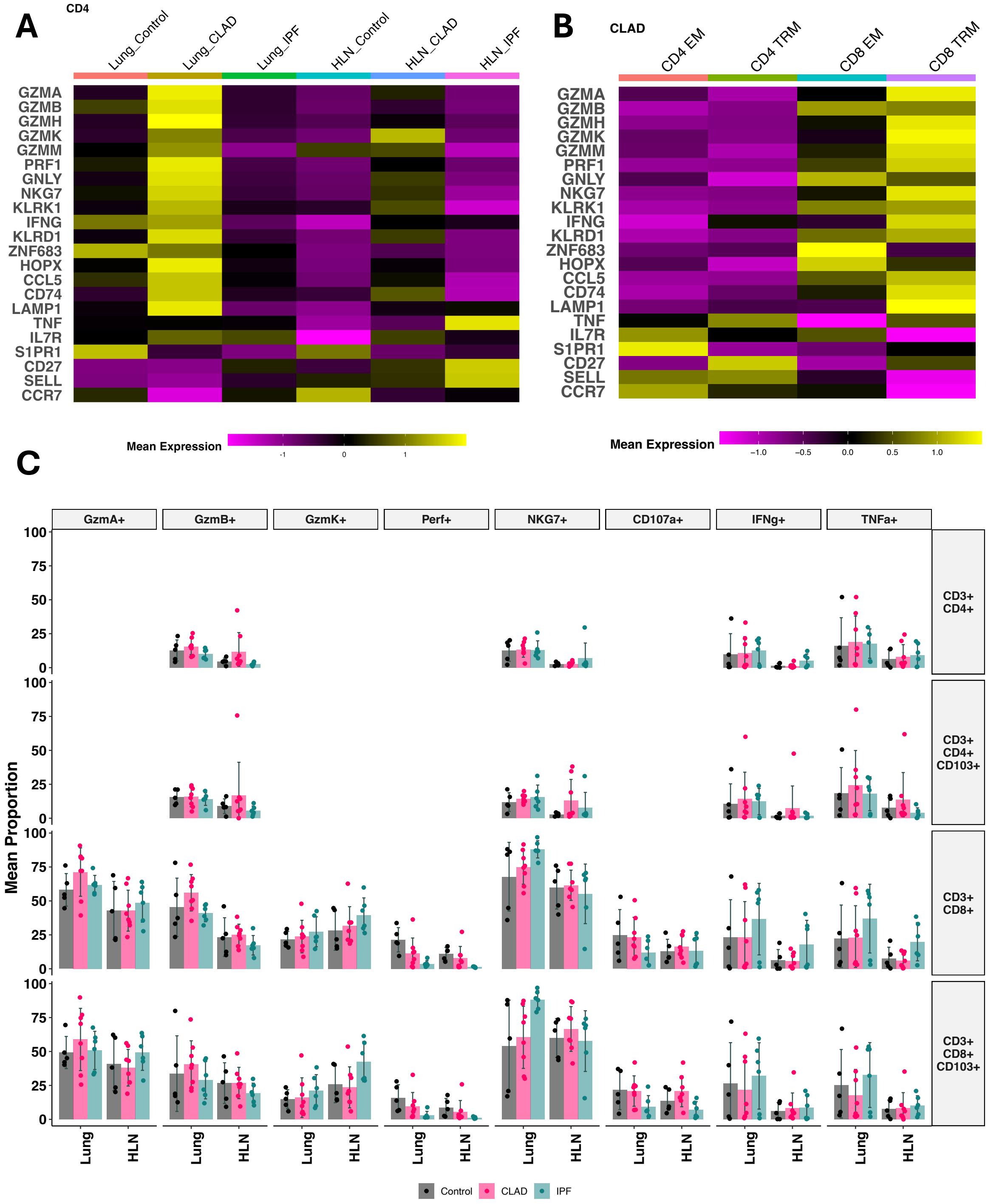

Figure S5: T cell cytokine transcript and protein expression levels. A) Mean normalized CD4+ T cell gene expression of genes associated with cytotoxic effector function by disease and anatomic location. B) Mean normalized CLAD T cell gene

expression by phenotype. C) Mean proportion of protein expression as measured via flow cytometry for all CD4+ and CD8+ T cells and their resident memory subsets.

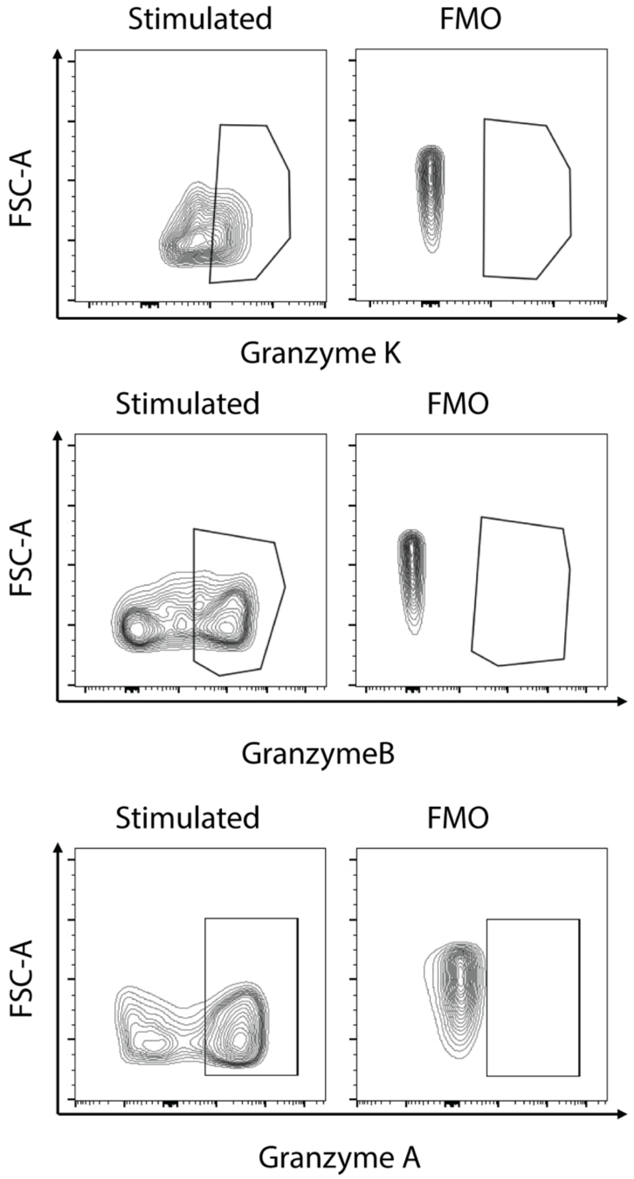

S6. Representative flow cytometry plots defining serine protease positivity based on fluorescence minus one (FMO).

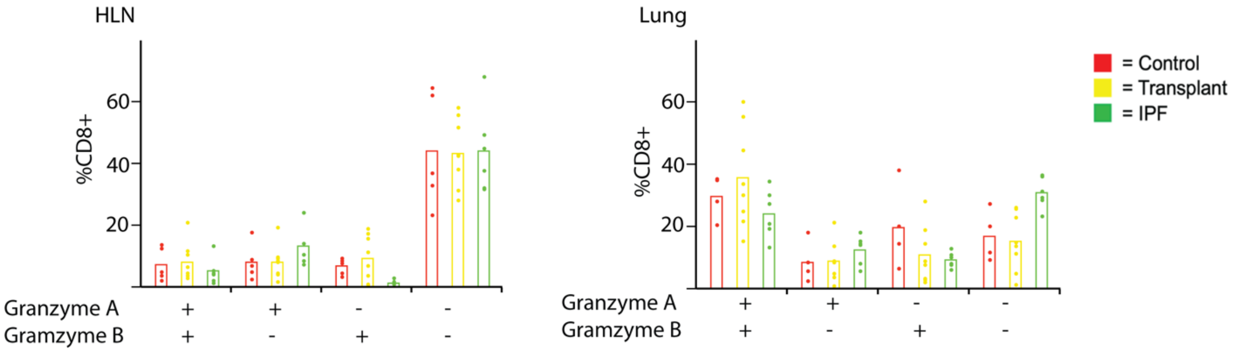

S7. Mean proportion of CD8^{+} T cells co-expressing Granzymes A and B in the HLN and Lung of lungs obtained from organ donors without underlying lung disease (control), idiopathic pulmonary fibrosis (IPF), and CLAD (Transplant). No statistically significant difference between conditions.

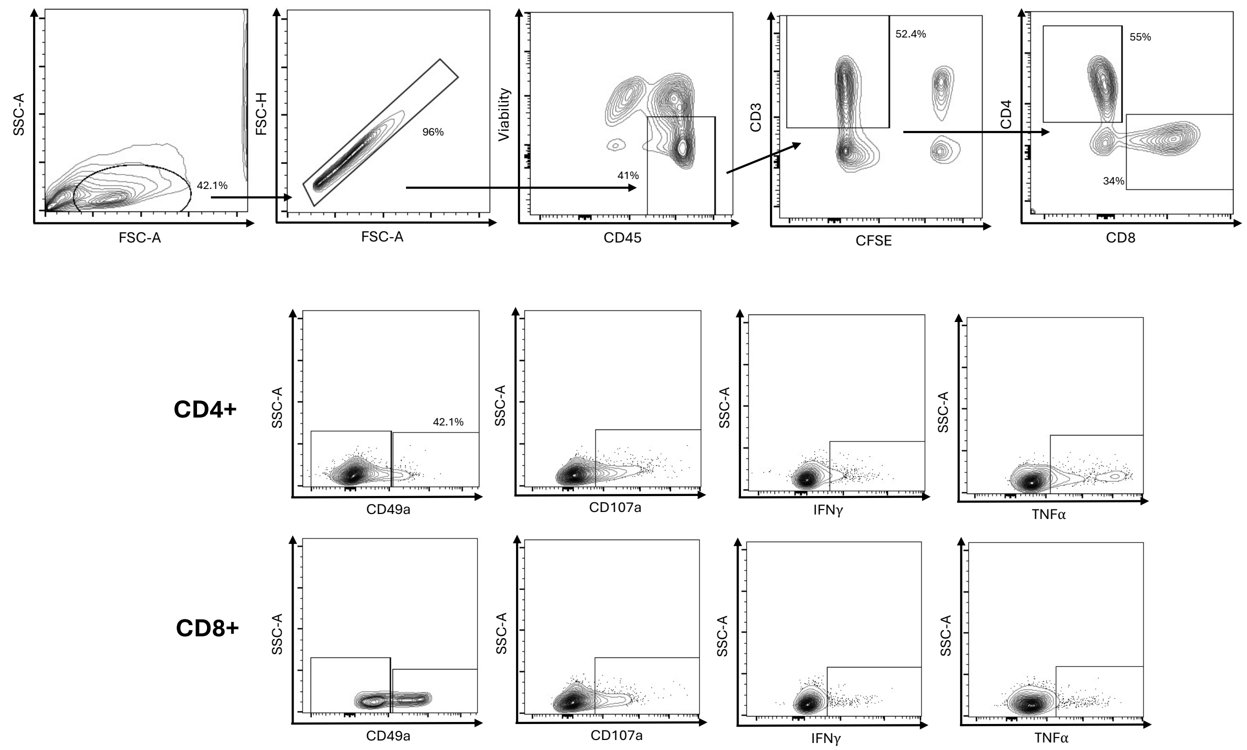

S8. Representative flow cytometry gating strategy for T cells obtained from murine grafts.

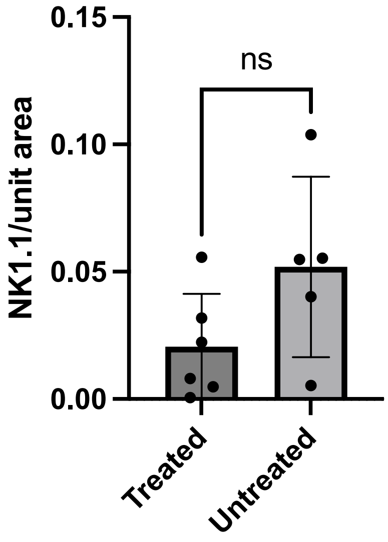

Figure S9: NK cell quantification following in-vivo NKG2D blockade. NK cells were quantified in the lung of orthotopically transplanted mice who were treated with NKG2D blockade compared to controls.

Supplementary Table 1: Tissue donor characteristics

TX_ID	Study_ID	Age	Gender	Diagnosis	Induction	CMV (D/R)	Experiment
2020-47-LT	CLAD1	24	Male	CLAD	Basiliximab	(-/+)	SC, IF
2021-10-LT	CLAD2	29	Female	CLAD	Alemtuzumab	(+/-)	SC, IF
2022-127-WA	CLAD3	78	Female	CLAD	Alemtuzumab	(+/-)	FC, IF
2020-45-LT	CLAD4	33	Female	CLAD	Basiliximab	(+/-)	FC, SC
2022-94-LT	CLAD5	37	Male	CLAD	Alemtuzumab	(-/+)	FC
2020-29-LT	CLAD6	27	Female	CLAD	Basiliximab	(+/-)	FC, IF
2019-16-LT	CLAD7	22	Female	CLAD	Alemtuzumab	(+/-)	IF
2022-96-WA	CLAD8	63	Female	CLAD	Basiliximab	(+/-)	FC
2022-125-LT	CLAD9	27	Female	CLAD	Alemtuzumab	(-/-)	FC
2023-92-LT	CLAD10	30	Female	CLAD			IF
2020-56-C	Control1	29	Female	Non-Diseased	NA	NA	FC, SC
2020-5-C	Control2	29	Male	Non-Diseased	NA	(+)	SC, IF
2020-60-C	Control3	27	Male	Non-Diseased	NA	(+)	FC
2019-24-C	Control4	68	Female	Non-Diseased	NA	(+)	FC
2020-61-C	Control5	28	Male	Non-Diseased	NA	(-)	FC
2019-28-C	Control6	29	Male	Non-Diseased	NA	NA	FC
2019-21-C	Control7	51	Female	Non-Diseased	NA	(-)	IF
2020-12-C	Control8	58	Male	Non-Diseased	NA	(-)	IF
2021-39-C	Control9	50	Male	Non-Diseased	NA	(-)	IF
2021-38-C	Control10	25	Male	Non-Diseased	NA	NA	SC
2021-41-C	Control11	81	Female	Non-Diseased	NA	NA	IF
2022-54-C	Control12	21	Male	Non-Diseased	NA	NA	IF
2023-15-C	Control13	23	Female	Non-Diseased	NA	NA	IF
2023-82-C	Control14	46	Male	Non-Diseased	NA	NA	IF
2023-66-C	Control15	57	Male	Non-Diseased	NA	NA	IF
2022-09-LT	IPF1	57	Female	IPF	NA	(+)	FC, SC
2022-13-LT	IPF2	68	Female	IPF	NA	(+)	FC, SC
2022-07-LT	IPF3	63	Male	IPF	NA	(+)	FC, SC
2022-15-LT	IPF4	68	Male	IPF	NA	(-)	FC, SC
2021-59-LT	IPF5	57	Male	IPF	NA	(-)	FC, SC
2021-48-LT	IPF6	69	Male	IPF	NA	(+)	FC, SC

IF: Immunofluorescence, SC: Single cell RNA/TCR, FC: flow cytometry, NA: Not available
CLAD: Chronic Lung Allograft Dysfunction, IPF: Idiopathic Pulmonary Fibrosis

Supplementary Table 2: Flow cytometry antibodies

Target	Clone	Species reactivity	Manufacturer	Fluorophore
**CD3**	SK7	Human	BD Biosciences	BUV 395
**Viability**	---	Human	Biolegend	Zombie UV
**CD56**	NCAM16.2	Human	BD Biosciences	BUV661
**CD8**	SK1	Human	Biolegend	BUV737
**Granzyme B**	QA18A28	Human	Biolegend	BV421
**CD4**	SK3	Human	BD Biosciences	BV480
**Perforin**	dG9	Human	Biolegend	BV510
**CD107a**	H4A3	Human	Biolegend	BV605
**IL-10**	JES3-9D7	Human	BD Biosciences	BV650
**PD1**	EH12.2H7	Human	Biolegend	BV711
**CCR7**	G043H7	Human	Biolegend	BV785
**Pan HLA**	W6/32	Human	Biolegend	FITC
**HLA B7**	BB7.1	Human	Biolegend	PE
**HLA A2**	BB7.2	Human	Biolegend	PE
**HLA A3**	GAP.A3	Human	Biolegend	PE
**CD45**	HI30	Human	Biolegend	PE-Cy5
**Granzyme K**	GM26E7	Human	Biolegend	PE-Cy7
**TNFa**	Mab11	Human	Biolegend	APC
**Granzyme A**	CB9	Human	Biolegend	AF647
**Granzyme B**	QA18A28	Human	Biolegend	AF700
**IFNg**	B27	Human	BD Biosciences	APC-R700
**CD103**	B27	Human	BD Biosciences	APC-Cy7
**HLA-DR**	G46-6	Human	BD Biosciences	BUV615
**CD25**	BC96	Human	Biolegend	BV421
**CD20**	2H7	Human	Biolegend	BV510
**CD45RA**	HI100	Human	Biolegend	BV605
**CD69**	FN50	Human	Biolegend	BV650
**CD31**	WM59	Human	Biolegend	BV711
**CRTAM**	Cr24.1	Human	Biolegend	PerCP-Cy5.5
**NKG2D**	1D11	Human	BD Biosciences	PE-CF594
**Granulysin**	DH2	Human	Biolegend	PE-Cy7
**FOXP3**	236A/E7	Human	BD Biosciences	AF647
**CD56**	5.1H11	Human	Biolegend	AF700
**CD49a**	SR84	Human	BD Biosciences	BV711
**CFSE**	---	Human	Fisher Scientific	---
**CD107a**	H4A3	Human	Biolegend	PE
**CD3**	UCHT1	Human	Biolegend	APC
**CD45**	30-F11	Mouse	Biolegend	BV510

**CD4**	GK1.5	Mouse	Biolegend	BV605
**NK1.1**	PK136	Mouse	Biolegend	BV785
**CD62L**	MEL-14	Mouse	Biolegend	PE
**CD8a**	53-6.7	Mouse	Biolegend	PerCP-Cy5.5
**CD44**	IM7	Mouse	Biolegend	PE-Cy7
**Nkg2D**	CX5	Mouse	Biolegend	APC
**CD3**	17A2	Mouse	Biolegend	APC-Cy7
**CD69**	H1.2F3	Mouse	BD Biosciences	BV480
**CD49a**	Ha31/8	Mouse	BD Biosciences	BV711
**HLA A2**	---	Mouse	Biolegend	FITC
**Perforin**	S16009A	Mouse	Biolegend	PE-Dazzle 594
**CD19**	6D5	Mouse	Biolegend	PE-Cy5
**CD45 (tail vein)**	30-F11	Mouse	Biolegend	AF700
**Fc Block**	---	Mouse	Biolegend	--
**RNAscope™**				
**Target**	**Gene ID**	**Species**	**Supplier**	**Accession Number**
**Klrk1**	27007	Mouse	ACD	NM_033078.4
**Cd8a**	12525	Mouse	ACD	NM_009857.1
**Scgb1a1**	22287	Mouse	ACD	NM_011681.2
